# A Biomimetic Multifunctional Scaffold for Infectious Vertical Bone Augmentation

**DOI:** 10.1002/advs.202310292

**Published:** 2024-05-05

**Authors:** Yifan Zhang, Zixin Li, Houzuo Guo, Qibo Wang, Bowen Guo, Xi Jiang, Yishu Liu, Shengjie Cui, Zhengda Wu, Min Yu, Lisha Zhu, Liyuan Chen, Ning Du, Dan Luo, Ye Lin, Ping Di, Yan Liu

**Affiliations:** ^1^ Department of Oral Implantology National Center for Stomatology National Engineering Research Center of Oral Biomaterials and Digital Medical Devices Beijing Key Laboratory of Digital Stomatology Translational Research Center for Oro‐craniofacial Stem Cells and Systemic Health Peking University School and Hospital of Stomatology Beijing 100081 China; ^2^ Department of Stomatology Peking University People's Hospital Beijing 100044 PR China; ^3^ CAS Center for Excellence in Nanoscience Beijing Key Laboratory of Micro‐nano Energy and Sensor Beijing Institute of Nanoenergy and Nanosystems Chinese Academy of Sciences Beijing 101400 China; ^4^ Department of Stomatology Beijing Chao‐Yang Hospital of Capital Medical University Beijing 100020 China; ^5^ Department of General Dentistry Laboratory of Biomimetic Nanomaterials Peking University School and Hospital of Stomatology Beijing 100081 China; ^6^ Central Laboratory Department of Orthodontics, National Center for Stomatology,National Engineering Research Center of Oral Biomaterials and Digital Medical Devices Beijing Key Laboratory of Digital Stomatology,Translational Research Center for Oro‐craniofacial Stem Cells and Systemic Health Peking University School and Hospital of Stomatology Beijing 100081 China

**Keywords:** antibacterial, mineralized collagen, osseointegration, silver nanowires, vertical bone augmentation

## Abstract

The regenerative treatment of infectious vertical bone defects remains difficult and challenging today. Current clinical treatments are limited in their ability to control bacteria and infection, which is unfavorable for new bone formation and calls for a new type of material with excellent osteogenic and antibacterial properties. Here a multifunctional scaffold is synthesized that mimics natural bone nanostructures by incorporating silver nanowires into a hierarchical, intrafibrillar mineralized collagen matrix (IMC/AgNWs), to achieve the therapeutic goals of inhibiting bacterial activity and promoting infectious alveolar bone augmentation in rats and beagle dogs. An appropriate concentration of 0.5 mg mL^−1^ AgNWs is selected to balance biocompatibility and antibacterial properties. The achieved IMC/AgNWs exhibit a broad spectrum of antimicrobial properties against Gram‐negative *Porphyromonas gingivalis* and Gram‐positive *Streptococcus mutans*. When the IMC/AgNWs are cocultured with periodontal ligament stem cells, it possesses excellent osteoinductive activities under both non‐inflammatory and inflammatory conditions. By constructing a rat mandibular infected periodontal defect model, the IMC/AgNWs achieve a near‐complete healing through the canonical BMP/Smad signaling. Moreover, the IMC/AgNWs enhance vertical bone height and osseointegration in peri‐implantitis in beagle dogs, indicating the clinical translational potential of IMC/AgNWs for infectious vertical bone augmentation.

## Graphical abstract

This study introduces a multifunctional scaffold (IMC/AgNWs) that mimics natural bone nanostructures by incorporating silver nanowires into a hierarchical, intrafibrillar mineralized collagen matrix, to achieve the therapeutic goals of inhibiting bacterial activity and promoting infectious alveolar bone augmentation in rats and beagle dogs.

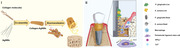

## 1 Introduction

Bone defect is common in the craniofacial region when it comes to numerous factors such as tumor, trauma, periodontal and peri‐implant diseases. Lack of horizontal and vertical bone could cause significant clinical issues and affect oral functions and facial appearance.^[^ ^1^ ^]^ Guided bone regeneration or onlay block transplantation could be an alternative way to build up these defects with high technical sensitivity.^[^ ^2^, ^3^ ^]^ However, it becomes more difficult to deal with infectious vertical bone loss, considering the inflammatory and bacterial environments, along with the maintenance of osteogenic space.

Peri‐implantitis, a typical infectious vertical bone defect, is a major biological complication that may lead to implant failure if left untreated. It is an inflammatory condition occurring in the tissues surrounding an osseointegrated implant, characterized by inflammation of the peri‐implant mucosa and progressive resorption of supporting bone.^[^ ^4^ ^]^ Studies have shown that the severity of bone loss due to peri‐implantitis increases with the duration of functional loading.^[^ ^5^ ^]^ Long‐term follow‐up studies spanning 5 to 10 years post‐implant placement found infection‐related implant loss in ≈20% of subjects.^[^ ^6^ ^]^ Evidence suggests techniques including bone grafting and membrane barriers may facilitate some degree of bone regeneration surrounding affected implants.^[^ ^6^, ^7^ ^]^ Historically, autogenous bone, allografts, xenografts, and synthetic grafts have been utilized.^[^ ^8^ ^]^ So far, there has been no consensus on the most effective grafting material.^[^ ^6^ ^]^ Given the difficulties of defect morphology, and bacterial and inflammatory environments, the optimum material for repairing infectious vertical bone defects must have strong mechanical support, antibacterial capabilities, and osteogenic properties.

Currently, progress has been achieved in the field of antimicrobial modification of bone implants, including the incorporation of antibiotics, organic antibacterial polymers, and particular immunoregulatory components.^[^ ^9^ ^]^ The use of synthetic polymers (such as polylactic acid) to load antibiotics or small molecule drugs is currently a common treatment mode because of its antibacterial ability, small inflammatory response, and degradability.^[^ ^10^ ^]^ However, it still faces problems such as a narrow antimicrobial spectrum, susceptibility to drug resistance, and poor osteoinductive properties of grafts. Another strategy is to use electroactive or photodynamic materials for sterilization. Although various types of materials have made significant progress in the field of bone tissue regeneration, from antibacterial, promoting cell proliferation, osteogenic differentiation to promoting bone regeneration, the operability and applicability of electroactive and photodynamic bone repair materials still need to be improved.^[^ ^11^, ^12^ ^]^

By replicating the physiochemical properties of natural bone extracellular matrix, biomimetic mineralized collagen scaffolds have created novel opportunities for regenerative defect healing. Our previous work has demonstrated effective synthesis of intrafibrillarly mineralized collagen (IMC) showcasing a native bone‐inspired hierarchical organization through a thermodynamically regulated self‐assembly approach.^[^ ^13^, ^14^, ^15^ ^]^ Compared to traditional mineralized collagen, the IMC provides a bone‐like microenvironment, which is conducive to the recruitment and osteogenic differentiation of endogenous stem cells, ultimately promoting in situ bone regeneration.^[^ ^16^, ^17^ ^]^ Endowing antibacterial properties to the IMC will help expand its application in infectious bone regeneration.

Nanosilver has garnered significant research attention recently as an efficient antibacterial agent. Previous studies have demonstrated that silver ions exert antibacterial effects by disrupting bacterial cell membrane transport, attaching and deforming DNA, and inactivating enzymes.^[^ ^18^ ^]^ Benefiting from size‐dependent properties, silver at the nanoscale possesses an enlarged specific surface area and altered surface electronic configuration relative to ions, overcoming silver ion limitations such as susceptibility to oxidation and precipitation. These advantages enhance its antibacterial performance and prolong efficacy.^[^ ^19^, ^20^ ^]^ Silver nanowires (AgNWs), which have been approved by the Food and Drug Administration, exhibit excellent antibacterial capability and stability, high conductivity, and flexibility.^[^ ^21^ ^]^ Compared to silver nanoparticles, AgNWs have a larger length‐to‐diameter ratio and a slower release of silver ions,^[^ ^22^ ^]^ resulting in lower toxicity to mammals but higher toxicity to microorganisms.^[^ ^23^ ^]^ In the present study, AgNWs were incorporated into a hierarchical, intrafibrillarly mineralized collagen matrix (IMC/AgNWs) to synthesize a novel bionic scaffold for regenerative treatment of peri‐implantitis lesions. The IMC/AgNWs exhibited a broad spectrum of antimicrobial properties and excellent osteoinductive activities even under inflammatory conditions in vitro. By constructing infectious vertical alveolar bone defect models in rats and beagle dogs, we showed that the IMC/AgNWs achieved a near‐complete healing and osseointegration by promoting osteogenesis and inhibiting bacteria‐induced inflammation.

## 2 Results

### 2.1 Fabrication and Characterization of IMC/AgNWs

**Figure 1:**
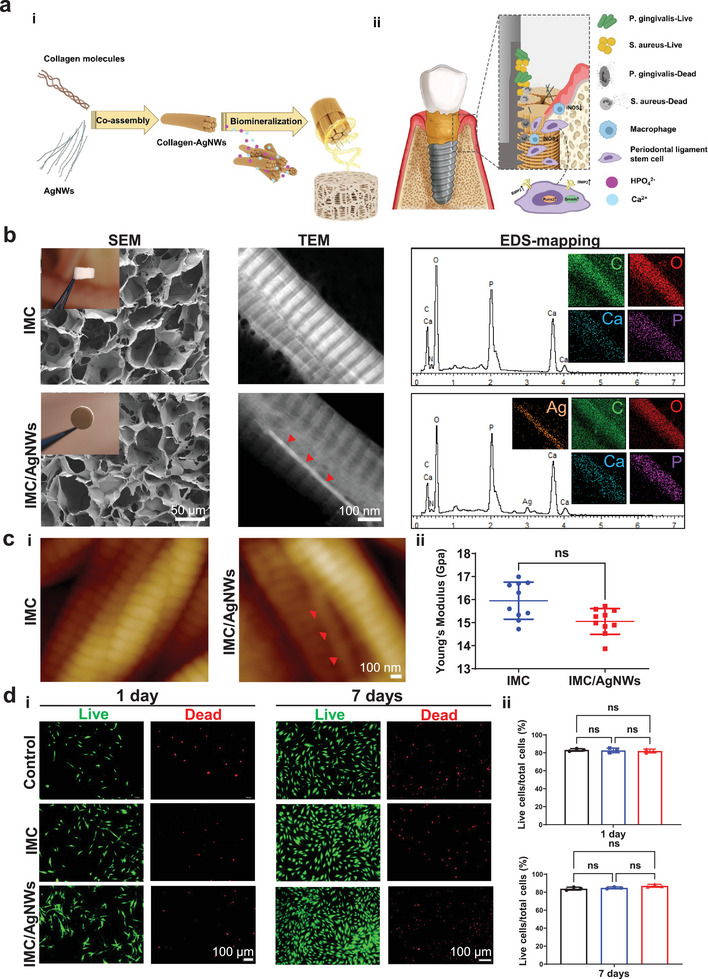
Physicochemical characterization and biocompatibility of IMC/AgNWs. a) (i) Schematic diagram of the assembly of IMC/AgNWs and (ii) the regenerative process of peri‐implantitis by IMC/AgNWs in beagle dogs. b) SEM, TEM, and EDS element mapping of IMC/AgNWs. Inset in SEM images: general views of scaffolds. The silver element could be clearly identified in EDS curves and mapping. c) (i) AFM nanostructures and (ii) Young's modulus of IMC/AgNWs. *n* = 10 independent samples. d) (i) Live/dead cytotoxicity assay of human PDLSCs cultured on IMC/AgNWs with the concentration of 0.5 mg mL^−1^ AgNWs. (ii) Semi‐quantitation of (i). *n* = 3 independent samples; ns: non‐significant difference. Red arrows: AgNWs assembled inside the mineralized collagen fibril.

An ideal scaffold for the effective treatment of infectious bone defects requires good biocompatibility, broad‐spectrum antibacterial activity, and excellent osteogenesis (**Figure** **1a**). Here, the multifunctional biomimetic bone substitute, IMC/AgNWs, was synthesized by intrafibrillar mineralization of the co‐assembled AgNWs with collagen molecules.^[^ ^24^ ^]^ Scanning electron microscopy (SEM) showed that both the IMC/AgNWs and IMC had a similar porosity of ≈91.09 ± 1.28% and interconnected pores of 117.12 ± 25.87 µm in diameter (Figure 1b). At the nanoscale level, the AgNWs could be successfully assembled into the IMC, as shown by transmission electron microscopy (TEM). The assembled IMC/AgNWs exhibited a bone‐like, hierarchically staggered nanostructure, similar to the IMC. Energy‐dispersive X‐ray spectroscopy (EDS) mapping coupled to TEM further showed that the element content of IMC/AgNWs was mainly composed of organic C and O, and inorganic Ca, P, and Ag, while the IMC only contained C, O, Ca, and P. The mass content of Ag was ≈5.77% in the IMC/AgNWs (Figure 1c). Atomic force microscopy (AFM) images revealed a bone‐like *D‐periodic* banding of IMC/AgNWs and IMC fibrils, consistent with TEM observations (Figure 1d). Semi‐quantitation showed that both IMC/AgNWs and IMC possessed similar Young's modulus of 15.05 ± 0.56 Gpa, comparable to that of natural alveolar bone.^[^ ^17^ ^]^

To systematically optimize AgNW concentration supporting cell proliferation and osteogenic differentiation in vitro, periodontal ligament stem cells (PDLSCs) were seeded on culture dishes pre‐coated with IMC/AgNWs fibrils incorporating varying AgNWs amounts (0, 0.125, 0.25, 0.5 and 1.0 mg mL^−1^). Cell Counting Kit‐8 (CCK‐8) assay determined 0.5 mg mL^−1^ of AgNWs maximized cell viability. Proliferation was enhanced versus the control (culture dishes) and IMC scaffold alone. However, the concentration at 1.0 mg mL^−1^ of AgNWs significantly decreased cell viability (Figure S1, Supporting Information). Therefore, 0.5 mg mL^−1^ of AgNWs was selected for subsequent experiments. Live/dead staining validated cell survival on IMC and IMC/AgNWs (0.5 mg mL^−1^ AgNWs) scaffolds after 1 and 7 days (Figure 1e,f). After 1 day of culture, 82.03 ± 2.16% of PDLSCs were alive in the IMC/AgNWs group, comparable to the IMC (82.70 ± 2.60%) and control (83.27 ± 1.45%) groups. Similarly, after 7 days, 87.03 ± 1.64% of PDLSCs survived in the IMC/AgNWs group, comparable to the IMC (84.80 ± 1.05%) and control (83.90 ± 1.60%) groups, confirming this concentration of 0.5 mg mL^−1^ AgNWs safely supporting cell growth and proliferation (Figure 1d).

### 2.2 Antibacterial Activities of IMC/AgNWs In Vitro

**Figure 2:**
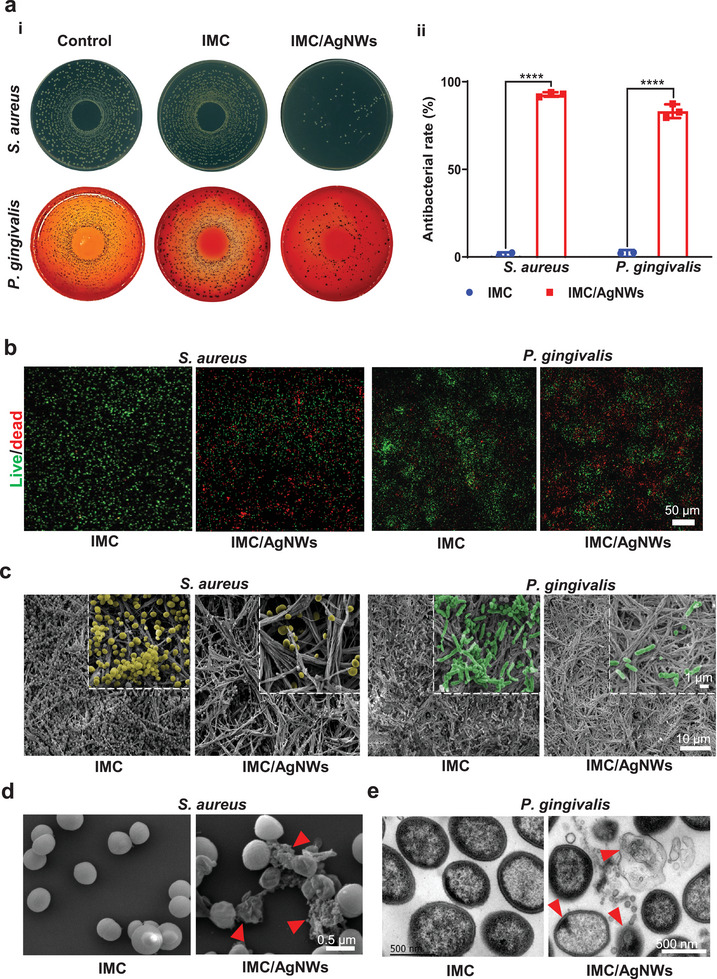
The antibacterial ability of IMC/AgNWs in vitro. a) Colony forming unit (CFU) assay of supernatant of *S. aureus* (24 h) and *P. gingivalis* (48 h) cultured on IMC and IMC/AgNWs. Control: culture plates. (i) Representative spreading plates. (ii) The semi‐quantitation of antibacterial rate for *S. aureus* (≈92.74%) and *P. gingivalis* (≈83.22%). b) Live/dead staining of the bacterial biofilm of *S. aureus* (24 h) and *P. gingivalis* (72 h) cultured on IMC and IMC/AgNWs for 24 h. Scale bar = 50 µm. c) Representative SEM images of the micro‐morphology of *S. aureus* (yellow, 24 h) and *P. gingivalis* (green, 72 h) cultured on IMC and IMC/AgNWs. d) Representative SEM images of supernatant of *S. aureus* cultured with IMC and IMC/AgNWs (right) for 24 h. The membranes in the IMC/AgNWs group were broken and dissolved (red arrows). Scale bar = 1 µm. e) Representative TEM images of supernatant of *P. gingivalis* cultured with IMC and IMC/AgNWs for 48 h. Red arrows: ultrastructural disruption of *P. gingivalis*. *n* = 3 independent samples; ^****^ *p*< 0.0001 versus IMC.

To investigate whether IMC/AgNWs have a broad spectrum of antimicrobial properties, we used two main microorganisms, Gram‐positive *Streptococcus mutans (S. aureus) and* Gram‐negative *Porphyromonas gingivalis (P. gingivalis)*, that cause common oral diseases such as peri‐implantitis and periodontitis. According to colony form units (CFU) assay by detecting upper suspensions of bacteria (**Figure** **2a**), the antibacterial rates of IMC/AgNWs (0.5 mg mL^−1^ AgNWs) against *S. aureus* and *P. gingivalis* were ≈92.74% (*p* < 0.0001 vs IMC) and ≈ 83.22% (*p*<0.0001 vs IMC), respectively. When the concentration of AgNWs increased to 1.0 mg mL^−1^, no bacteria were observed (Figure S2a, Supporting Information). Confocal imaging of the live/dead staining revealed higher contents of dead bacteria with both *S. aureus* and *P. gingivalis* cultured on the surface of IMC/AgNWs (0.5 mg mL^−1^ AgNWs), compared to the IMC. (Figure 2b). SEM were further used to observe bacteria in direct contact with scaffolds (Figure 2c). Compared to the IMC group, the number of bacterial colonies was significantly higher than that in the IMC/AgNWs group (0.5 mg mL^−1^ AgNWs). Bacteria on the IMC surface maintained intact cellular morphology and gathered to form biofilms, similar to those in the control group (culture plates, Figure S2b, Supporting Information). By contrast, the bacterial number was greatly reduced as well as the appearance of the bacteria was extraordinarily distorted and incomplete in the contrast in the IMC/AgNWs group. Furthermore, Ag nanoparticles could be detected around the damaged bacteria. To further understand the antibacterial mechanism of IMC/AgNWs, the rupture of cellular membrane was observed by detecting the top suspensions of bacteria. *S. aureus* in the IMC group exhibited round morphology with a complete membrane, while damaged *S. aureus* cellular membranes were observed in the IMC/AgNWs group (Figure 2d). TEM visualized *P. gingivalis* ultrastructural disruption following IMC/AgNWs treatment, including decreased electron density and empty cell walls (Figure 2e; Figure S2c, Supporting Information). Collectively, IMC/AgNWs potently inhibited the growth and viability of both *P. gingivalis* and *S. aureus*.

### 2.3 Osteoinductive Activities of IMC/AgNWs under Non‐Inflammatory or Inflammatory Conditions

**Figure 3:**
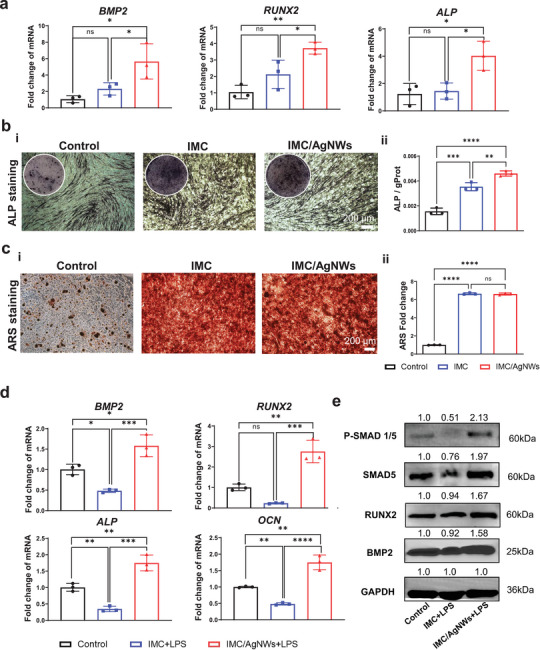
Osteoinductive activity of IMC/AgNWs under non‐inflammatory or inflammatory conditions. a) mRNA expression levels of osteogenic differentiation‐related genes including *BMP2*, *RUNX2*, and *ALP* in human PDLSCs cultured on 6‐well plate (Control), IMC, and IMC/AgNWs for 7 days. b) ALP staining (i) and semiquantitative analysis (ii) of human PDLSCs cultured on 48‐well plate, IMC, and IMC/AgNWs for 7 days. c) ARS staining (i) and semiquantitative analysis (ii) of human PDLSCs cultured on 6‐well plate (Control), IMC, and IMC/AgNWs for 7 days. f) mRNA expression levels of osteogenic differentiation‐related genes including *BMP2*, *RUNX2*, *ALP, and OCN* in human PDLSCs cultured on 6‐well plate (Control), IMC with LPS treatment (IMC+LPS) and IMC/AgNWs with LPS treatment (IMC/AgNWs+LPS) for 7 days. d) Western blotting of BMP2, RUNX2, SMAD5, and *p*‐SMAD1/5 in human PDLSCs cultured on 6‐well plate (Control), IMC+LPS, and IMC/AgNWs +LPS at 7 days. *n* = 3 independent samples; ^*^: *p*< 0.05; ^**^: *p* < 0.01; ^***^: *p* < 0.001; ^****^: *p*< 0.0001.

To assess the osteoinductivity of IMC/AgNWs in vitro, quantitative RT‐PCR, alkaline phosphatase (ALP), and Alizarin Red S staining were performed and 0.5 mg mL^−1^ AgNWs was used for further biological experiments. After 7 days of osteogenic induction, mRNA expression of bone morphogenetic protein 2 (BMP2, *p*< 0.05 vs control and *p*<0.05 vs IMC), runt‐related transcription factor‐2 (RUNX2, *p*< 0.01 vs control and *p*<0.05 vs IMC), and ALP (*p*< 0.05 vs control and *p*<0.05 vs IMC) associated with osteogenesis, increased substantially in PDLSCs cultured on IMC/AgNWs (**Figure** **3a**). Compared to the control and IMC groups, PDLSCs in the IMC/AgNWs group demonstrated elevated ALP expression (*p*< 0.0001 vs control and *p*<0.01 vs IMC, Figure 3b,c) and mineralized nodule formation, visualized qualitatively and quantitatively (*p*< 0.0001 vs control, Figure 3d,e).

Osteoinduction under inflammation induced by 10 µg mL^−1^ *P. gingivalis*‐LPS was also evaluated. Quantitative RT‐PCR revealed that significantly upregulated *BMP2* (*p*< 0.05 vs control and *p*<0.001 vs IMC), *RUNX2* (*p*< 0.01 vs control and *p*<0.001 vs IMC), osteocalcin *(OCN, p*< 0.01 vs control and *p*<0.0001 vs IMC), and *ALP* (*p*< 0.01 vs control and *p*<0.001 vs IMC) mRNA in PDLSCs cultured on IMC/AgNWs after 7 days of incubation (Figure 3d). The trend of western blotting was consistent with the expression trend of mRNA results. The protein expression levels of BMP2, RUNX2, drosophila mothers against decapentaplegic protein 5 (SMAD5) and phosphorylation of SMAD5 (P‐Smad1/5) were significantly increased in the IMC/AgNWs group compared to the control and IMC groups (*p*< 0.01, Figure 3e; Figure S3, Supporting Information), suggesting that the BMP2/Smad/Runx2 pathway may be implicated in osteogenic differentiation under inflammatory conditions. Collectively, IMC/AgNWs effectively promoted osteogenic differentiation of PDLSCs in normal and inflammatory microenvironments.

To examine the in vitro anti‐inflammatory ability of IMC/AgNWs, 1 µg mL^−1^ *P. gingivalis*‐LPS was added to the culture medium and mouse mononuclear macrophages  RAW264.7 were cultured on different substrates. After 24 h of culture, the mRNA expression of inflammatory markers including interleukin‐6 (*Il6*, *p*< 0.0001), inducible nitric oxide synthase (*Inos*, *p*< 0.01), and tumor necrosis factor‐alpha (*Tnfα*, *p*< 0.05) was remarkedly reduced in the IMC/AgNWs group compared to the IMC and control groups. After 72 h of culture, the mRNA expression of anti‐inflammatory markers in the IMC/AgNWs group was significantly higher than that in other groups, including mannose receptor (*Cd206*, *p*< 0.01), interleukin‐10 (*Il10*, *p*< 0.05), and arginase‐1 (*Arg1*, *p*< 0.05) (Figure S4a, Supporting Information). Besides, immunofluorescence showed higher intensity fluorescence expression of CD206 (*p*< 0.01) and lower intensity fluorescence expression of iNOS (*p*< 0.05) in the IMC/AgNWs group compared to the IMC and control groups (Figure S4b, Supporting Information). These results revealed that the IMC/AgNWs scaffold provided an anti‐inflammatory microenvironment.

### 2.4 In Vivo Application of IMC/AgNWs on Rat Infected Periodontal Defects

**Figure 4:**
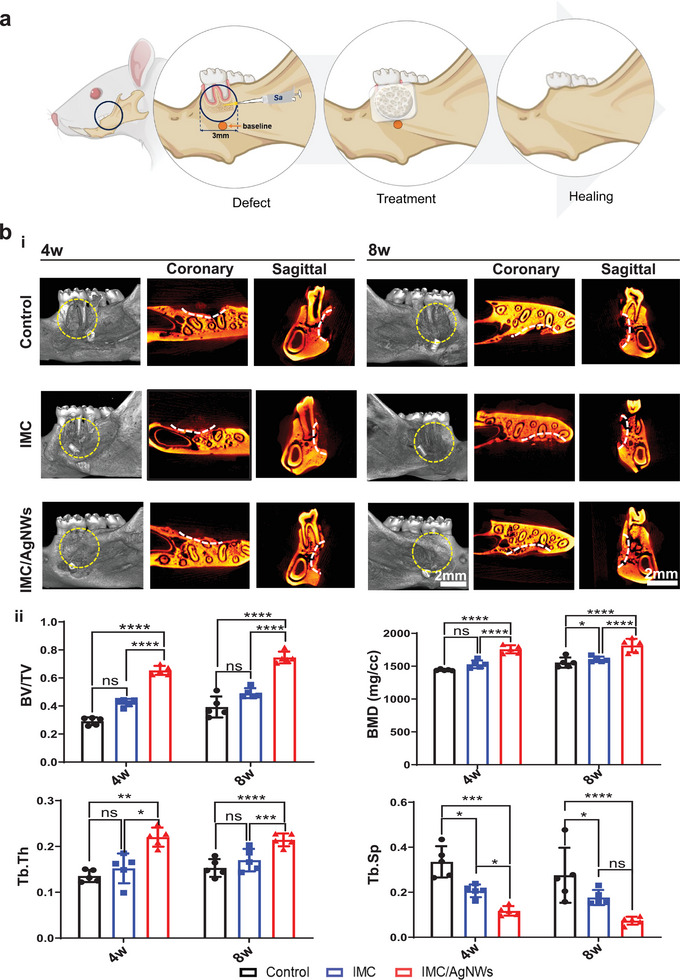
In vivo application of IMC/AgNWs on rat infected periodontal defects. a) Schematic diagram of the healing process of bacteria‐infected periodontal defects in rats by IMC/AgNWs. b) Representative micro‐CT images of the infected engineered bones in different groups after 4 and 8 weeks of implantation (i). Control: without any implants. Gutta‐percha: baseline; white lines: defect boundary in both coronary and sagittal sections. Yellow dotted circle: defect area. (ii) Quantitative analysis of BV/TV/, BMD, Tb. Th, and Tb. Sp of newly formed alveolar bone in micro‐CT images (*n* = 5 biologically independent samples, by one‐way ANOVA with Tukey's post hoc test. ^*^: *p*< 0.05; ^**^: *p* < 0.01; ^***^: *p* < 0.001; ^****^: *p*< 0.0001. ns: non‐significant difference.

A rat mandibular infectious periodontal defect model was established to confirm the antibacterial and bone‐formation performances of IMC/AgNWs in vivo (**Figure** **4a**). Critical‐sized periodontal bone defects (1.5 mm in depth, 3 mm in diameter) were prepared, and *S. aureus* suspensions (10 µL, 1 × 10^7^ CFU mL^−1^) were injected to the defects, and then the IMC/AgNWs (0.5 mg mL^−1^ AgNWs) and IMC were implanted into the defect areas. The Bio‐Gide^®^ membrane was used to cover the defects (Figure S5a, Supporting Information).

The efficiency of IMC/AgNWs in the regeneration therapy of infected periodontal defects was validated by comparing the volume and quality of new bone in the negative control group (using Bio‐Gide^®^ membrane only), and experimental groups (using IMC or IMC/AgNWs covered with Bio‐Gide^®^ membrane). Micro‐CT 2D and 3D imaging revealed alveolar bone regeneration around the defect in the IMC/AgNWs group at 4 weeks postoperatively and was nearly complete at 8 weeks (Figure 4b). There was less low‐density new bone formation in the IMC group, with minimal new bone observed in the control group at week 8. Quantitative micro‐CT analysis (Figure 4c) confirmed enhanced bone regeneration based on new bone volume fraction (BV/TV), bone mineral density (BMD), trabecular thickness (Tb. Th), and spacing (Tb. Sp) in the IMC/AgNWs group compared to others. 4 weeks after implantation, most of the defect area in the IMC/AgNWs group was filled with new bone (65.38 ± 3.21%), which was much more than that in the IMC group (42.41 ± 2.62%, *p*< 0.0001) and the control group (29.12 ± 2.78%, *p*< 0.0001). The BMD in the IMC/AgNWs group (1757.11 ± 61.65 mg cc^−1^) was higher than that of the IMC group (1528.83 ± 59.23 mg cc^−1^, *p*< 0.0001) and the control group (1441.967 ± 11.63 mg cc^−1^, *p*< 0.0001). The same tendency could be observed in the changes in trabecular structural parameters Tb. Th (*p*< 0.01 vs control and *p*< 0.05 vs IMC) and Tb. Sp (*p*< 0.0001 vs control and *p*< 0.001 vs IMC). As the implantation time extended to 8 weeks, the quantitative micro‐CT analysis showed that most of the bone defect was healed (74.61 ± 4.16%) by the IMC/AgNWs, whereas 49.15 ± 3.57% of the defect area was repaired in the IMC group, and less new bone was formed (39.32 ± 7.59%) in the control group (*p*< 0.0001 vs control and IMC groups). In addition, the BMD in the IMC/AgNWs group (1815.78 ± 102.44 mg cc^−1^) was higher than that in the IMC group (1603.98 ± 42.13 mg cc^−1^, *p*< 0.0001) and the control group (1555.29 ± 77.60 mg cc^−1^, *p*< 0.0001). Similarly, the bone trabecular parameters of Tb. Th (*p*< 0.01 vs control and *p*< 0.05 vs IMC) and Tb. Sp (*p*< 0.01 vs control and *p*< 0.05 vs IMC) in the IMC/AgNWs group showed better results.

**Figure 5:**
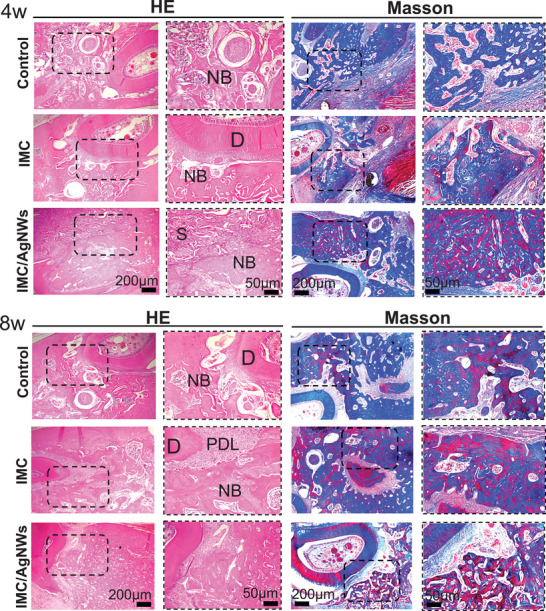
Representative HE and Masson's trichrome staining images of the infected engineered bones in different groups after 4 and 8 weeks of implantation. D: dentin, PDL: periodontal ligament, NB: newly formed bone. S: scaffold.

The microstructure of the regenerated tissues at 4 and 8 weeks was investigated using HE and Masson's trichrome staining (**Figure** **5**). Compared to the IMC and control groups, the IMC/AgNWs group regenerated greater new bone tissue formation with lesser inflammatory cell infiltration. Masson's trichrome staining demonstrated denser, mature new bone (darker red) in the IMC/AgNWs group than other groups, consistent with micro‐CT findings. To specifically evaluate the histological results, a modified Histological Scoring System was further introduced.^[^ ^25^, ^26^ ^]^ We evaluated the tissue response after material implantation from five aspects: union, cancellous bone, cortical bone, bone marrow of bone regeneration and inflammation (**Table** **1**). At weeks 4 and 8, the IMC/AgNWs group had significantly higher scores than the control group in terms of union, cortical bone, arrow, and inflation (*p* < 0.05). In addition, the scores of the IMC/AgNWs group at 4 and 8 weeks of inflammation were significantly higher than those of the IMC group (**Table** **2**). No biotoxicity of IMC/AgNWs was found in the HE staining of internal organs (Figure S5b, Supporting Information).

**Table 1:** Histological Scoring System (modified).

Item	Score
Union	
No evidence of union	0
Fibrous union	1
Osteochondral union	2
Bone union	3
Complete organization of shaft	4
Cancellous bone	
No osseous cellular activity	0
Early apposition of new bone	1
Active apposition of new bone	2
Reorganizing cancellous bone	3
Completely reorganizing cancellous bone	4
Cortical bone	
None	0
Early appearance	1
Formation under way	2
Mostly reorganized	3
Completely formed	4
Marrow	
None in resected area	0
Beginning to appear	1
Present in more than half of the defect	2
Complete colonization by red marrow	3
Mature fatty marrow	4
Inflammation	
Large confluent areas with inflammatory cells present	0
Several large conglomerates of inflammatory cells present	1
Several small conglomerates of cells present	2
One or more small conglomerates of cells present	3
A few single cells present	4
Total (Maximum)	20

**Table 2:** Histological score.

	Median (Minimum‐Maximum), *n* = 5					
	Control		IMC		IMC/AgNWs	
	4 w	8 w	4 w	8 w	4 w	8 w
Union	1(0‐1)	1(1‐2)	2(1‐2)	2(2‐3)	2(2‐3) ^a)^	4(3‐4) ^[Link]^, ^[Link]^
Cancellous bone	1(0‐1)	2(1‐2)	1(1‐2)	2(2‐3)	2(1‐3)	3(2‐4) ^a)^
Cortical bone	0(0‐0)	1(0‐2)	1(0‐1)	2(1‐3)	1(1‐2) ^a)^	3(2‐3) ^a)^
Marrow	0(0‐1)	1(1‐2)	1(1‐2)	2(2‐3)	2(1‐2) ^a)^	3(2‐3) ^a)^
Inflammation	0(0‐0)	2(1‐2)	1(0‐1)	2(2‐3)	2(1‐2) ^[Link]^, ^[Link]^	4(3‐4) ^[Link]^, ^[Link]^

^a)^ Compare to group Control, *p* < 0.05;

^b)^ Compare to group IMC, *p* < 0.05; Kruskal‐Wallis nonparametric ANOVA.

Since the degradation behavior of scaffolds is closely related to the regeneration effect, the degradation rate of IMC/AgNWs was evaluated both in vitro and in vivo. According to the in vitro degradation results, the IMC/AgNWs degraded to 92.53%, 72.66%, 17.10%, and 5.23% the remaining weight in collagenase degradation solution on days 1, 3, 5 and 7, while the remnant IMC was 90.97%, 73.64%, 20.55%, and 3.47% on days 1, 3, 5 and 7 (Figure S6a, Supporting Information). There was no significant difference between the two groups at any time points. SEM images (Figure S6b, Supporting Information) demonstrated significant structural damage and morphological changes of IMC/AgNWs and IMC in collagenase degradation solution over time. The in vivo degradation rate was calculated based on HE staining tissue sections of rat infected periodontal defects at 4 and 8 weeks (Figure S6c, Supporting Information). The results showed that the remnant IMC/AgNWs was ≈ 26.73% at 4 weeks of implantation, and decreased to ≈ 9.67% at the 8 weeks. Similar results were found in the IMC group (Figure S6c, Supporting Information).

**Figure 6:**
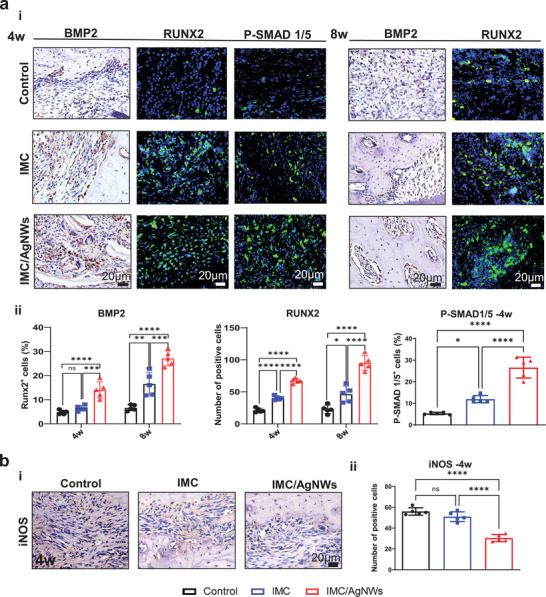
a) Representative Immunohistochemical and immunofluorescent staining images of BMP2, RUNX2, and P‐SMAD 1/5 in the defect areas at different time points (i). (ii) Semi‐quantitative analysis of positive cell numbers in (i). b) Representative Immunohistochemical staining images of iNOS (i) and (ii) semi‐quantitative analysis of positive cell numbers at 4 weeks in the defect areas. *n* = 5 biologically independent samples, by one‐way ANOVA with Tukey's post hoc test. ^*^: *p*< 0.05; ^**^: *p* < 0.01; ^***^: *p* < 0.001; ^****^: *p*< 0.0001. ns: non‐significant difference.

To further validate if the BMP2/Smad/Runx2 pathway participated in the regeneration process, immunohistochemistry and immunofluorescence staining were carried out. Immunofluorescence showed elevated, concentrated RUNX2 expression in the IMC/AgNWs group (14.00% ± 1.52 at 4 w, 27.12% ± 1.39 at 8 w) compared to the IMC group (6.40% ± 0.51 at 4 w, 16.47% ± 2.14 at 8 w, *p* < 0.0001) and control group (4.80% ± 0.37 at 4 w, 6.61% ± 0.55 at 8 w, *p* < 0.0001) (**Figure** **6a**). P‐SMAD1/5 upregulation showed a consistent trend among groups at 4 weeks (IMC/AgNWs: 26.56% ± 2.16; IMC: 11.94% ± 0.75, *p* < 0.0001; Control: 5.31 ± 0.24, *p* < 0.0001). Immunohistochemistry revealed comparative BMP2 expression kinetics (Figure 6a) (IMC/AgNWs: 67.20 ± 1.66/slice at 4 w and 95.00 ± 5.15/slice at 8 w; IMC: 40.20 ± 1.39/slice at 4 w and 46.20 ± 5.56/slice at 8 w, *p* < 0.001; Control: 21.20 ± 1.46/slice at 4 w and 22.60 ± 2.89/slice at 8 w, *p* < 0.0001). Overall, the data support IMC/AgNWs activation of the BMP2/Smad/Runx2 pathway during regeneration. Furthermore, iNOS expression was lower in the IMC/AgNW group at 4 weeks, suggesting an anti‐inflammatory effect (30.40 ± 1.54 per slice compared to 51.00 ± 2.07 for IMC, *p* < 0.0001 and 56.00 ± 1.58 for control, *p* < 0.0001, Figure 6b). These findings were consistent with in vitro results.

### 2.5 IMC/AgNWs Regenerates Peri‐Implant Alveolar Defects in Beagle Dogs

**Figure 7:**
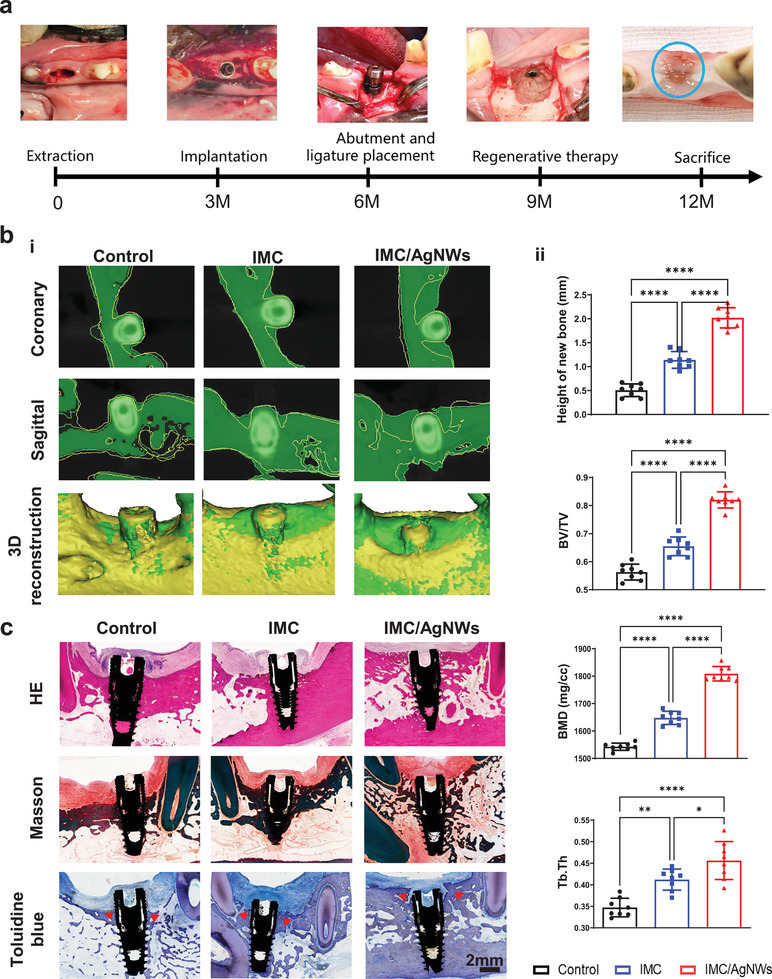
IMC/AgNWs regenerates peri‐implant alveolar defects in beagle dogs. a) Schematic outline and surgical process of peri‐implantitis in beagle dogs. b) Representative coronary and sagittal sections, and 3D reconstruction of peri‐implantitis areas (i). On both coronary and sagittal CBCT, the yellow line outlines the bone resorption profile before treatment, while the green areas beyond the yellow line are the new bone after treatment. Similarly, the green parts represent the new bone in the peri‐implantitis area in 3D reconstruction model. (ii) Quantitative analysis of new bone height, BV/TV/, BMD, and Tb:Th of newly formed alveolar bone in micro‐CT images. d) Representative HE, Masson, and toluidine blue staining images of newly formed bone (red arrows) among three groups. (*n* = 5 biologically independent samples, by one‐way ANOVA with Tukey's post hoc test ^*^: *p* < 0.05; ^**^: *p* < 0.01; ^***^: *p* < 0.001; ^****^: *p* < 0.0001.

The beagle peri‐implantitis model was established using the standardized technique of ligating surgical thread around implant fixtures, a classic mature method that induces pathology comparable to clinical conditions,^[^ ^27^, ^28^, ^29^ ^]^ which had great value to the clinical treatment. It took 1 year to induce and treat peri‐implantitis in 6 beagles with 24 implant sites total (**Figure** **7a**; Figure S7a–d, Supporting Information). Cone beam computed tomography (CBCT) was performed after osseointegration (6 months), pre‐treatment (9 months), and post‐regenerative therapy (12 months) to intuitively observe peri‐implant bone formation (Figure S7e, Supporting Information). Registration of the two CT scans identified bone regeneration after therapy (Figure 7b). The IMC/AgNWs group exhibited more new bone outlined in both sagittal and coronal sections (green area external to the yellow line).

Micro‐CT statistics revealed significantly greater vertical new bone height in the IMC/AgNWs group (2.021 ± 0.211 mm) versus the IMC (1.140 ± 0.175 mm, *p* < 0.0001) and control groups (0.507 ± 0.132 mm, *p* < 0.0001) (Figure 7c). IMC/AgNWs also exhibited obviously higher new alveolar BV/TV (82.0 ± 2.9%) compared to IMC (65.5 ± 3.3%, *p* < 0.0001) and controls (0.563 ± 0.028, *p* < 0.0001). The BMD in the IMC/AgNWs group (1808.73 ± 26.39 mg cc^−1^) was higher than that in the IMC group (1647.76 ± 23.91 mg cc^−1^, *p* < 0.0001) and the control group (1542.81 ± 13.18 mg cc^−1^, *p* < 0.0001). Similarly, the Tb. Th which reflects trabecular bone parameters of new alveolar bone was significantly greater in the IMC/AgNWs group than that of the other two groups (*p* < 0.0001 vs control and *p* < 0.05 vs IMC). Likewise, HE, Masson, and toluidine blue staining (new bone indicated by red arrows) demonstrated excellent osteogenesis and near‐complete healing of the experimental defect (Figure 7d). No remnant IMC or IMC/AgNWs was found after 12 weeks of implantation from the histological staining section, indicating that both IMC and IMC/AgNWs could be completely degraded in vivo up to 3 months.

## 3 Discussion

Currently, bone repair materials used for infectious vertical bone regenerative therapy mainly include antibiotic bone cement, biodegradable antibiotic sustained‐release systems, electroactive and photodynamic materials. Antibiotic combined with Polymethyl methacrylate or calcium phosphate cement is currently a sustained‐release bone graft material for bone tissue infections, but their non‐degradability limits their clinical application.^[^ ^30^ ^]^ Ceramic calcium phosphate (such as hydroxyapatite, and β‐tricalcium phosphate) and synthetic polymers (such as Polylactic acid) are other common carriers for antibiotics. These systems have the characteristics of high initial release and long duration. However, compared with natural bone, these grafts lack osteoinduction and have poor physicochemical, biological, and degradability properties, posing greater risks of bone resorption.^[^ ^31^ ^]^

Implants with drug‐laden coatings achieve short‐term anti‐inflammatory and antibacterial effects in preventing plaque colonization.^[^ ^32^ ^]^ However, antibiotics tend to induce bacterial resistance and fail to maintain  effective drug concentrations for long‐term. Nano‐modifying titanium implant surfaces^[^ ^33^, ^34^ ^]^ also faced challenges like weak corrosion resistance, susceptibility to aging, and incompatibility between osteogenic and antibacterial effects. For cases with obvious vertical bone defects from periodontitis or peri‐implantitis, soft tissue pressure could inhibit bone regeneration without a mechanically strong stent or scaffolds.^[^ ^35^ ^]^ Collectively, improved regenerative approaches are needed to address these limitations.

In this study, we fabricated a biomimetic, bio‐functional scaffold by assembling AgNWs within mineralized collagen fibrils, mimicking native bone mechanical properties. Rather than surface deposition, AgNWs were wrapped inside IMC to establish a biocompatible tissue interface while retaining antimicrobial functions. The IMC/AgNWs exhibited a broad spectrum of antimicrobial properties and excellent osteoinductive activities even under inflammatory conditions in vitro. By constructing infectious vertical alveolar bone defect models in rats and beagle dogs, we showed that the IMC/AgNWs achieved a near‐complete healing and osseointegration by promoting osteogenesis and inhibiting bacteria‐induced inflammation.

Similar to the etiology of periodontitis, clinical and animal experiments have confirmed that plaque biofilm is the important cause of peri‐implantitis and other infectious bone defects.^[^ ^36^ ^]^ Current clinical treatments including mechanical and chemical debridement provide weak and unsustainable antiinfection and antibacterial effects, as the inflammatory environment is unfavorable for osteogenesis.^[^ ^37^ ^]^ This study used *S. aureus* and *P. gingivalis* as model bacteria, as they are the two most common pathogenic bacteria associated with oral infectious disease representing the aerobic Gram‐positive and anaerobic Gram‐negative phenotypes, respectively.^[^ ^38^, ^39^ ^]^ In vitro antibacterial assays demonstrated that 0.5 mg mL^−1^ AgNWs could destroy bacterial cellular structures and disturb biofilms. Higher AgNWs concentrations correlated with stronger antibacterial activity. The antimicrobial mechanism of nano‐silver relies mainly on three aspects: nano‐effects, silver ion antimicrobial action, and reactive oxygen species generation. Compared to bulk silver, nanoscale silver has a larger surface area overcoming the disadvantages of oxidization and precipitation of silver ions, providing longer antibacterial efficacy and performance.^[^ ^19^, ^20^ ^]^ The specific electrical structure of silver nanomaterials can disrupt bacterial cell membranes and walls.^[^ ^40^ ^]^ Concurrently, oxidation of nano‐silver releases antimicrobial silver ions that bind DNA, inducing deformation, inhibiting transit across bacterial membranes, producing oxidative stress, and deactivating enzymes.^[^ ^41^, ^42^ ^]^ While biotoxicity concerns arise from possible large releases of free silver ions,^[^ ^43^ ^]^ relevant studies confirm silver nanomaterial safety.^[^ ^44^, ^45^ ^]^ Additionally, limited contact between nanomaterials and the body, as well as silver nanoparticles' minimal toxicity to organisms as a whole, have been shown.^[^ ^46^, ^47^ ^]^ In this study, AgNWs demonstrate better biocompatibility and lower toxicity both in vitro and in vivo. When the concentration of AgNWs increased to 1.0 mg mL^−1^, more discrete Ag nanoparticles were observed, accompanied by a sharp decrease in measured biocompatibility. Therefore, 0.5 mg mL^−1^ of AgNWs was used and produced excellent antibacterial, anti‐inflammatory and in situ bone formation effects.

The BMP signaling pathway classically regulates bone formation. The Smad1/5 signaling pathway can be activated by BMP2, inducing downstream Runx2 expression as revealed by previous researches.^[^ ^48^, ^49^ ^]^ Similarly, Liu et al. showed that promotion of mesenchymal stem cell osteogenesis was mainly mediated by activation of the BMP2/Smad5 pathway.^[^ ^50^ ^]^ In our study, obvious upregulation of SMAD5 and P‐SMAD1/5 expression was found in the IMC and IMC/AgNWs groups under inflammatory conditions in vitro. Furthermore, increased expression of P‐SMAD1/5 was again observed after IMC/AgNWs treatment of infected periodontal defects in rats. Based on these results, promotion of PDLSC osteogenesis under inflammatory conditions may be mediated by activation of the BMP2/Smad/Runx2 pathway. The immune mechanism of antibacterial and anti‐infectious effects of IMC/AgNWs needs to be explored further.

Among different clinical challenges, peri‐implantitis and periodontal defects are typical infected vertical bone defects and are a major biological complication that may lead to implant failure if not treated promptly. To mimic clinical settings, we assessed the translational potential of IMC/AgNWs in two models: infected periodontal defects in rats and peri‐implantitis in beagles. The peri‐implantitis model established in this study is comparable to clinical conditions, and has important value for clinical treatment. Owing to their balanced osteogenic and antibacterial properties, IMC/AgNWs achieved excellent regeneration in repairing both defects. Comparison with the control group showed that extensive inflammatory cell infiltration persisted after debridement alone or in combination with IMC, highlighting the importance of the antimicrobial and anti‐inflammatory properties of IMC/AgNWs. Histological evaluation and imaging analyses indicate the possibility of applying IMC/AgNWs to treat peri‐implantitis and infectious vertical bone defects. Our future study needs to involve a wider variety of bacteria and include long‐term efficacy assessments.

## 4 Conclusion

In summary, a biomimetic multifunctional scaffold was constructed for infectious vertical bone augmentation in rats and beagle dogs. The bone‐like staggered nanostructure and mechanical properties of IMC/AgNWs endowed the scaffold with excellent biocompatibility and osteoinduction. Furthermore, the IMC/AgNWs possessed a broad spectrum of antimicrobial properties and anti‐inflammatory function, owing to a larger length‐to‐diameter ratio and a slower release of silver ions of AgNWs assemble inside the scaffold. Therefore, self‐assembly and biomimetic mineralization technologies can be widely used to synthesize multifunctional scaffolds for complex bone defect repair.

## 5 Experimental Section

### Preparation of Silver Nanowires (AgNWs)

0.334 g of polyvinylpyrrolidone powder was dissolved in 17 mL of ethylene glycol and added to the flask, and then the mixture was heated to 170 °C. 0.025 g of silver chloride powder was dissolved in 1 mL of ethylene glycol and injected into the polyvinylpyrrolidone solution at 170 °C. After 5 min of reaction, the prepared ethylene glycol solution of silver nitrate (0.110 g, 2 mL) was injected into the reaction flask within 10 min. The reaction was maintained at 170 °C for 30 min, during which magnetic stirring was continued. After the reaction was completed, the mixture was naturally cooled to room temperature, an appropriate amount of water and ethanol were added, and then centrifuged at 8000 r min^−1^ for 5 min to collect the precipitate to remove the solvent. The obtained precipitate was dispersed again in a mixed solvent of water and ethanol, and then centrifuged at 1000 r min^−1^ for 15 min to collect the precipitate. The above experiment was repeated three times. The obtained AgNWs were dispersed in water for subsequent characterization and applications.

### Synthesis of IMC/AgNWs

A biomimetic self‐assembly method was used to synthesize the IMC/AgNWs scaffold.^[^ ^14^, ^15^, ^16^ ^]^ Briefly, pre‐fabricated AgNWs (10 mg mL^−1^) were mixed with type I tropocollagen (Corning, 4.05 mg mL^−1^) and reconstituted in a potassium buffer containing 30 mm Na_2_HPO_4_, 200 mm KCl, and 10 mm KH_2_PO_4_ for 48 h at 37 °C. The assembled collagen was cross‐linked using 0.3 m 1‐ethyl‐3‐(3‐dimethylaminopropyl)‐carbodiimide and 0.06 m N‐hydroxysuccinimide for 4 h and mineralized using 9 mm CaCl_2_, 4.2 mm K_2_HPO_4_ and 0.2 mg mL^−1^ poly‐aspartic acid for at 37 °C for 7 days. To prepare bone‐like porous scaffolds, the assembled collagen was frozen at −20 °C overnight and lyophilized for 12 h. To fabricate the IMC and IMC/AgNWs on culture plates for SEM, biocapacity, and antibacterial tests, 1 mL of 1 mg mL^−1^ type I tropocollagen solution or the mixture (collagen: AgNWs = 1:19) was dripped onto the bottom of the well and left to gel by incubation at 37 °C for 2 days. Finally, the assembled collagen was crosslinked for 4 h and mineralized at 37 °C for 3 days (Table S1, Supporting Information).

### Scanning Electron Microscopy (SEM)

The microstructure of scaffolds was observed by SEM (Hitachi SU8020, Japan for scaffolds) at 15 kV. The bacteria and biofilms were first fixed with 2.5% glutaraldehyde 0.1 m phosphate buffer for 15 min, being observed by field emission scanning electron microscope (FE‐SEM, JEOL JSM‐7900F, Japan for bacteria). All the samples were dehydrated in a graded sequence of ethanol (50%−100%), dried to the critical point, and then coated with gold using a sputtering process for 2 min at 20 mA.

### Transmission Electron Microscopy (TEM)

The ultrastructure of scaffolds and bacteria was examined by TEM at 100 kV. The IMC and IMC/AgNWs fibrils were deposited onto Formvar carbon‐coated nickel grids and stained with 1% sodium phosphotungstate for TEM imaging (Hitachi HT7700, Japan). The bacteria and biofilms were first fixed with 2.5% glutaraldehyde 0.1 m phosphate buffer for 1 h, followed by staining in 1% OsO_4_, dehydration in ethanol (30 to 100%) and embedded in epoxy resin at 65 °C for 48 h. Ultra‐thin sections of 70–100 nm thickness were obtained using an ultramicrotome, collected on copper grids, and observed by TEM (JEOL JEM‐1011).

### Atomic Force Microscopy (AFM)

Atomic force microscopy (AFM) was used to assess the nanomechanical characteristics of the scaffolds (Dimension Icon, Bruker, USA). Individual layers of IMC and IMC/AgNWs were constructed on freshly cleaved mica surfaces under ambient conditions (room temperature). For each specimen, three scanned representative regions were analyzed to determine the Young's modulus. Fifteen random samples measuring 600×600 nm were selected from each mapping image. Nano Scope 18 software evaluated the 512×512‐pixel property maps obtained from the scans. This yielded evaluation of the Young's modulus for both scaffold types at the nanoscale level.

### Human Periodontal Ligament Stem Cell (PDLSC) Isolation and Culture

Human periodontal ligament stem cells (PDLSCs) were isolated as previously described.^[^ ^51^ ^]^ In brief, periodontal ligament tissue was scraped from tooth roots and digested for 1 h at 37 °C in a solution containing 4 mg mL^−1^ dispase II (Solarbio) and 3 mg mL^−1^ type I collagenase (Sigma). Single‐cell suspensions were then used to establish PDLSC cultures. The obtained PDLSCs were employed to assess biocompatibility, proliferation, and osteogenic induction potential of the scaffolds. All experimental protocols were approved by the Ethical Committee of Peking University School of Stomatology (No. PKUSSIRB‐201951179).

### Cell Counting Kit‐8 Assay

The 48‐well plates precoated with IMC or IMC/AgNWs at varying concentrations were sterilized by immersion in 75% ethanol for 2 h, followed by exposure to ultraviolet light for 2 h prior to use. The 48‐well plates were used as controls. Identified human PDLSCs (1×10^4^) were seeded onto the different films on days 1, 3, and 7. Cell viability was assessed on each day using a microplate reader to measure optical density values at 450 nm, following the manufacturer's instructions for the quantification assay.

### Live/Dead Cytotoxicity Assay

Identified human PDLSCs (1×10^4^) were seeded onto the different substrates as previously described on days 1 and 7. For live/dead staining, a working solution comprising 2 µm calcein acetoxymethyl and 8 µm propidium iodide was prepared following the manufacturer's instructions. After gently washing adherent cells with PBS to remove the supernatant, cells were incubated with the working solution at room temperature for 30–45 min. Subsequently, the staining solution was aspirated, and the incubation terminated. A total of 10 µL of PBS was added to transfer stained cells to a clean glass slide, which was then covered and sealed. Labeled cells were observed under a fluorescence microscope to assess viability.

### Bacteria Strain and Culture Conditions

*Porphyromonas gingivalis* (*P. gingivalis, ATCC 33277*) was routinely incubated at 37 °C in an anaerobic chamber, containing an atmosphere of 80% N_2_ and 20% CO_2_. The liquid media of brain‐hear infusion broth (BHIS) was supplemented with hemin (5 mg mL^−1^) and vitamin K1(1 µg mL^−1^). The solid media of BHIS blood agar was acquired after adding 20 mg mL^−1^ brain‐heart infusion agar (Oxoid Inc., Ogdensburg, NY, USA) and 50 µL mL^−1^ sterile defibrinated sheep blood to the liquid media. Gram staining and 16S rDNA identification were performed to confirm pure culture status of *P. gingivalis ATCC 33277. Staphylococcus aureus* (*S. aureus*) *26003* was cultured in a Luria‐Bertani (LB) medium containing tryptone (10 g L^−1^), yeast extract (5 g L^−1^), and NaCl (10 g L^−1^) at 37°C overnight. The solid culture medium was added 20 mg mL^−1^ agar (Oxoid Inc., Ogdensburg, NY, USA).

### Colony Form Units (CFU) Assay

*S. aureus* and *P. gingivalis* were diluted to 1×10^6^ CFU mL^−1^ and inoculated on different substrates (control, IMC, and 0.5 mg mL^−1^‐IMC/AgNWs) for 24 and 48 h in a 48‐well plate. The upper suspensions were sucked out, mixed, and serially diluted. All the samples were inoculated on sterile agar plates for 24 (*S. aureus*) and 72 h (*P. gingivalis*) for further counting by using easy‐spiral Pro (Interscience, St. Nom La Brete`che, France).

### Live/dead Fluorescent Staining

*S. aureus* (1×10^6^ CFU mL^−1^) and *P. gingivalis* (1×10^7^ CFU mL^−1^) were inoculated on different substrates (IMC, 0.5 mg mL^−1^‐IMC/AgNWs) as previously described. After being rinsed 3 times with 0.9% saline, the biofilms were stained with LIVE/DEAD Baclight bacterial viability kit (Invitrogen, Waltham, MA, USA) for 20 min. The stained biofilms were then observed by a confocal laser scanning microscope (Leica Microsystems, Mannheim, Germany). Excitation and emission wavelengths were set to be 488 and 520 nm, respectively.

### SEM Imaging of the Biofilm and *S. aureus*

*S. aureus* (1×10^6^ CFU mL^−1^) and *P. gingivalis* (1×10^7^ CFU mL^−1^) were inoculated on different substrates (IMC, 0.5 mg mL^−1^‐IMC/AgNWs) as previously described. After being washed with PBS and fixed with 2.5% glutaraldehyde 0.1 m phosphate buffer for 15 min, followed by serial dehydration with ethanol, the biofilms were examined under a field emission scanning electron microscope (FE‐SEM, JEOL JSM‐7900F, Japan). The upper suspensions of treated *S. aureus* were centrifugated and resuspended in 2.5% glutaraldehyde 0.1 m phosphate buffer for 15 min, followed by serial dehydration. A liquid‐drop was mounted onto aluminum stubs and examined under a FE‐SEM.

### TEM Imaging of *P. gingivalis*

*P. gingivalis* (1×10^7^ CFU mL^−1^) was inoculated on different groups of films (IMC, 0.5 mg mL^−1^‐IMC/AgNWs) for 48 h in a 48‐well plate. The upper suspensions were sucked out and centrifugated. The sediment was fixed in 2.5% glutaraldehyde 0.1 m phosphate buffer for 1 h, followed by staining in 1% OsO4, dehydration in ethanol (30‐100%) and embedment in epoxy resin polymerized at 65 °C for 48 h. The samples were sectioned with an ultramicrotome collected on copper grids and examined under a transmission electron microscope (JEOL JEM‐1011).

### Alkaline Phosphatase Staining

For alkaline phosphatase (ALP) assay, identified human PDLSCs (1×10^4^) were cultured in osteogenic medium (culture media supplemented with 0.05 mm ascorbic acid 2‐phosphates, 10 mm β‐glycerophosphate, and 10^−7^ m dexamethasone) on different substrates (control, IMC, 0.5 mg mL^−1^‐IMC/AgNWs) for 7 days. ALP activities of PDLSCs in the different groups were measured with Alkaline Phosphatase Assay Kit (Beyotime, China) following the manufacturer's protocol. After incubation with p‐nitrophenyl phosphate, the quantitative ALP activity was measured by measuring the OD values at 405 nm.

### Alizarin Red S Staining

To detect mineral nodule formation, identified human PDLSCs (1×10^6^) were fixed and stained with 2% Alizarin Red S (ARS) (Sigma–Aldrich, USA) solution after culturing in osteogenic medium for 14 days. The photographs were captured. The stained cells were then detained with 10% acetylpyridinium chloride (Sigma‐Aldrich, USA) and measured for OD values at 590 nm.

### Quantitative Real‐Time Polymerase Chain Reaction (RT‐PCR)

Identified human PDLSCs (5×10^3^) were cultured in osteogenic medium on different substrates (control, IMC, 0.5 mg mL^−1^‐IMC/AgNWs) as previously described for 7 days. For IMC+LPS and IMC/AgNWs+LPS groups, PDLSCs (5×10^3^) were cultured in osteogenic media supplemented with lipopolysaccharide (10 µg mL^−1^ *P. gingivalis*‐LPS, Sigma‐Aldrich, USA) for 7 days to evaluate osteogenic activity under inflammatory conditions. Total RNA was extracted using Trizol reagent (Thermo Fisher Scientific), and cDNA was synthesized using SuperScript III One‐Step RT‐PCR System with Platinum Taq High Fidelity (Invitrogen). Quantitative RT‐PCR was performed on a 7900HT Fast Real‐Time PCR using SYBR Green (Invitrogen). The primers were listed in Table S2 (Supporting Information).^[^ ^50^ ^]^

### Western Blotting

Identified human PDLSCs (5×10^3^) in IMC+LPS group and IMC/AgNWs+LPS group were cultured in LPS induced‐osteogenic medium for 7 days as previously described, while the osteogenic medium in control group did not contain LPS. Total proteins from cell lysates were harvested by RIPA Buffer (Thermo Fisher Scientific, Cat#89 900) with Protease/Phosphatase Inhibitor Cocktail (Thermo Fisher Scientific, Cat#87 786) on ice. The lysates underwent a 20‐min, 12000 × g centrifugation at 4 °C. Pierce BCA protein assay kit (Cat#23 225, Thermo Fisher Scientific) was used to quantify proteins. The absorbance was measured with a Bio‐Rad microplate reader at 595 nm. Then, equal amounts of 20–30 µg proteins were mixed with 5× loading buffer (Solarbio, P1040) and cooked for 10 min at 99 °C. The samples were separated by 10% SDS–polyacrylamide gel electrophoresis, and then transferred to polyvinylidene difluoride membranes and blocked in 5% nonfat milk in 0.1% TBS‐Tween (Sigma‐Aldrich, P9416). The membranes were separately probed with corresponding primary antibodies against GAPDH (1:1000, Proteintech, Cat#60004‐1‐lg), BMP2 (1:1000, Abcam, Cat#AB214821), Runx2(1:5000, CST, Cat#D1L7F), Smad5 (1:1000, CST, Cat#12 534) and *p*‐Smad1/5 (1:1000, CST, Cat#12 534) overnight at 4 °C. Membranes were washed three times in TBS with 0.1% Tween‐20 for 5–10 min each time, and HRP‐conjugated secondary antibodies (1:10 000, diluted with TBST, ZSGB‐BIO, Cat#ZB‐2301 and Cat#ZB‐2305) were incubated for 1 h, washed twice in TBS with 0.1% Tween‐20 and imaged by an Odyssey Imaging System. Quantitative analysis was performed by Image J software (Table S3, Supporting Information).

### The Effect of IMC/AgNWs on Inflammatory Responses In Vitro

Identified RAW264.7 cells were cultured in DMEM containing 10% FBS at 37 °C in 5% CO_2_ on different substrates (control, IMC, 0.5 mg mL^−1^‐IMC/AgNWs). After 6 h of adhesion, the culture medium of RAW264.7 cells was replaced with fresh DMEM containing 10% FBS and 1 µg mL^−1^ *P. gingivalis*‐LPS (Sigma–Aldrich, USA). After 24 and 72 h, total RNA from cultured RAW264.7 cells was isolated and used for quantitative real‐time RT‐PCR.

### Immunofluorescence Staining

Briefly, after 24 h of culture on differently coated coverslips, RAW264.7 cells were fixed in 4% paraformaldehyde, permeabilized with 0.25% Triton‐X, and blocked by 1% BSA. Subsequently, the primary antibodies against CD68 (Cat#28058‐1‐AP), CD206 (Cat#AB64693) and iNOS (Cat#AB178945) with 1:100 dilution were dropped onto coverslips and incubated overnight at 4 °C. Then the coverslips were incubated with the respective fluorescein isothiocyanate‐conjugated (Cat#ZF‐0311) or tetramethylrhodamine isothiocyanate‐conjugated (Cat# SR131) secondary antibody for 30 min, followed by 5 min of nuclear staining with DAPI. The samples were observed with a Zeiss laser‐scanning microscope (LSM 510) coupled to a LSM 5 release 4.2 software.

### In Vivo Application on Rat Infected Periodontal Defect Models

Infected periodontal defect models in rats were performed according to the previously published protocols^[^ ^17^ ^]^ (Figure 4a; Figure S2a, Supporting Information). Male Wistar rats (150–200 g, 6 weeks old) were randomly divided into three groups including the negative control, IMC, IMC/AgNWs groups (*n* = 5 for each group, Table S4, Supporting Information). After anesthetized and disinfected, the buccal alveolar bone, periodontal ligament, and root surface of the left mandibular first molar with round burs were removed and a hemispherical periodontal defect (1.5 mm in depth, 3 mm in diameter) was created. A tip of gutta‐percha was marked at the edge of the defect and 10 µL of *S. aureus* (1×10^7^ CFU mL^−1^) was injected into the defect area. For the control group, the defect area was filled without any scaffolds and covered with Bio‐Gide membrane only. For the experimental groups, the periodontal defect area was filled with IMC or 0.5 mg mL^−1^‐IMC/AgNWs scaffolds plus Bio‐Gide membrane respectively. Each site was implanted with the same size of cylindrical IMC or IMC/AgNWs scaffolds (4 mm in diameter, 2 mm in height). No seeded cells were used in either group. After 4 weeks, a similar infected‐hemispherical‐periodontal defect was created in the right side through the same approach. Four weeks later, rats were sacrificed by over‐anesthesia, and both mandibles were obtained and fixed with 10% formalin. All animal procedures were approved by the Peking University Institutional Animal Care and Use Committee (No. LA2021064).

### In Vivo Application on Beagle Peri‐Implantitis Models

Six 1‐year male beagles underwent extractions, implant placement, and ligature placement for induction of peri‐implantitis utilizing an expedited approach^[^ ^28^ ^]^ (Figure 7a). In detail, six beagles had their bilateral mandibular premolars P2 and P4 extracted, and were fed with liquid food for two weeks, then fed with normal food for 2.5 months. After 3 months of healing, 24 implants (3.4 mm in diameter and 9 mm in length) were implanted. During the next 3 month of implantation healing, normal feeding and oral plaque control with chlorhexidine cotton ball were required. At 6 months, the abutment and ligature were placed at the same time. After 3 months for induction of peri‐implantitis (Figure S4a, Supporting Information), the ligature was removed and an open flap debridement was performed to remove the granulation tissue and allow for further regenerative therapy (Figure S4b, Supporting Information). All the 24 sites were divided into 3 groups randomly. For the negative control group, nothing was put after debridement. For the experimental groups, the peri‐implant defect was filled with IMC or 0.5 mg mL^−1^‐IMC/AgNWs scaffolds (Figure S4c, Supporting Information). Each site was implanted with enough scaffolds (6 mm in diameter, 2 mm in height, 3–5 pieces) to fill up the defective area. Three months after regenerative treatment, all the beagles were sacrificed, and the mandibles were harvested (Figure S4d, Supporting Information). The animal research protocol was approved by the Peking University Institutional Animal Care and Use Committee (No. LA2021065).

### Microcomputed Tomographic (micro‐CT) Analysis

All fixed samples were scanned with a micro‐CT system (Inveon, Simens, German) at 80 kV and 500 µA. The Inveon Research Workplace 4.0 software was used for 3D image reconstruction. The region of interest area was selected semiautomatically, and the bone volume and thickness of the newly formed alveolar bone were measured by the software automatically (Table S5, Supporting Information).

### Histological Staining Assessment

The harvested samples were fixed immediately in 10% neutral buffered formalin for 24 h, dehydrated using a gradient ethanol (70–100%), and embedded in paraffin blocks. Histological sections with 5 µm in thickness were prepared using a microtome. HE, toluidine blue, and Masson's trichrome stainings were performed according to standard procedures. The protein expression in the tissues was examined using immunohistochemistry or immunofluorescence. All the sections underwent antigen retrieval using 0.125% trypsin and 20 µg mL^−1^ proteinase K for 30 min at 37 °C, followed by being blocked for 1 h with 5% bovine serum albumin (BSA, Solarbio, Cat# A8010). For immunohistochemistry, tissue sections were incubated with anti‐BMP2 (Abcam, Cat#AB214821) or anti‐iNOS (Abcam, Cat#AB178945) at a dilution of 1:200 by 3% w/v BSA overnight at 4 °C, followed by washing and incubating with horseradish‐peroxidase‐conjugated secondary antibodies (ZSGB‐BIO, diluted with PBS, Cat#PV‐9001). For immunofluorescence staining, sections were incubated with anti‐Runx2 (CST, Cat#D1L7F) or anti‐P‐SMAD 1/5 (CST, Cat#12 534) at a dilution of 1:100 by 3% w/v BSA overnight at 4 °C, followed by being incubated with FITC secondary antibodies (ZSGB‐BIO, diluted with PBS, Cat#ZF‐0311). Confocal microscopic images were acquired with a laser‐scanning microscope (LSM 510, Zeiss, Germany), and then processed with LSM 5 Release 4.2 software. Each group included a minimum of three slides, all of which were examined at the defect margin and the defect center including the scaffolds.

### Radiographic Evaluation in 3‐D for Peri‐Implantitis

All the dogs underwent periapical radiographs and CBCT scanning before and 3 months after the regenerative therapy, using the same projection condition (NewTom 5G XL, NewTom, Italy). The 2 sets of Dicom data (before regenerative therapy, and 3 months after the treatment) were output and transferred to volumetric imaging software (Mimics Research 20.0, Materialise, Leuven, Belgium), in which virtual models of the lower jaw were 3‐D reconstructed and superimposed.

### The Degradation Assay of IMC/AgNWs In Vitro and In Vivo

To evaluate the degradation rate of IMC/AgNWs in vitro, the scaffold was immersed in media (Dulbecco's PBS with MgCl_2_ and CaCl_2_, Sigma–Aldrich) containing 20 µg collagenase type I (0.1 mg mL^−1^, Sigma, USA) at 5% CO_2_, 95% humidity, and 37 °C with a fixed ratio of mass to medium volume of 0.1 mg mL^−1^ (ISO 10993–6:2016 [E]). The scaffolds were lyophilized and weighed on days 1, 3, 5 and 7 after immersion. The degradation rate is indicated by the rate of remnant weight of IMC/AgNWs using a formula below:

(1) $$\mathrm{The}\;\;\mathrm{rate}\;\mathrm{of}\;\;\mathrm{remnant}\;\mathrm{weight}\;=M_{t}\;/\;M_{0}\;\times 100\%$$

where, *M*_0_: The weight of scaffolds before immersion. *M*_t_: The weight of scaffolds at each immersion time point.

To evaluate the degradation rate of scaffolds in vivo, HE staining sections of rat infected periodontal defects after 4, and 8‐weeks treatment were used. The percentage of the retained scaffold area in the sections was calculated in image J software.

### Statistical Analysis

All experiments were performed in at least biological triplicates. Statistically significant differences were assessed by unpaired two‐tailed Student's *t*‐test and one‐way analysis of variance (ANOVA) with Turkey's post hoc test (among three groups) using GraphPad Prism 10 (GraphPad Software Inc.). *p*≤ 0.05 was considered statistically significant. ^*^ *p* ≤ 0.05; ^**^ *p* ≤ 0.01; ^***^ *p* ≤ 0.001.

## Conflict of Interest

The authors declare no conflict of interest.

## Author Contributions

Y.Z. and Z.L. contributed equally to this work. Y. L. and P.D. designed experiments, analyzed the data, revised the manuscript, and provided funding support. Y.‐F.Z. and Z.‐X.L. performed the experiments, analyzed the data, and performed the manuscript. B.‐W.G., Q.‐B.W., S.‐J.C., and M.Y. assisted with scaffold fabrication and characterization. H.‐Z.G., X.J., Y.‐S.L., and Z.‐D.W. assisted with animal experiments. Q.‐B.W., L.‐S.Z., and L.‐Y.C. performed some in vivo experiments. N.D. analyzed the micro‐CT and staining data in vivo. D.L. assisted with scaffold fabrication, and revised the manuscript. Y. L. revised the manuscript and provided funding support. All authors reviewed the manuscript.

## Supporting information

Supporting Information

## Data Availability

The data that support the findings of this study are available from the corresponding author upon reasonable request.

## References

[1] S. Abtahi , X. Chen , S. Shahabi , N. Nasiri , ACS Mater Au 2023, 3, 394. 38089090 10.1021/acsmaterialsau.3c00013 PMC10510521

[2] S. Li , J. Zhao , Y. Xie , T. Tian , T. Zhang , X. Cai , Int. J. Oral. Sci. 2021, 13, 37. 34782595 10.1038/s41368-021-00143-3 PMC8594427

[3] Nickenig, H. J. , J. E. Zöller , M. Kreppel , Periodontol. 2000, 93, 327. 10.1111/prd.12524 37940190

[4] F. Schwarz , G. Alcoforado , A. Guerrero , D. Jönsson , B. Klinge , N. Lang , N. Mattheos , B. Mertens , J. Pitta , A. Ramanauskaite , S. Sayardoust , I. Sanz‐Martin , A. Stavropoulos , L. Heitz‐Mayfield , Clin. Oral. Implants Res. 2021, 32, 245. 34642987 10.1111/clr.13827

[5] Monje, A. , J. Nart , Periodontol. 2000, 88, 182. 10.1111/prd.12418 35103326

[6] F. Khoury , P. L. Keeve , A. Ramanauskaite , F. Schwarz , K.‐T. Koo , A. Sculean , G. Romanos , Int. Dent. J. 2019, 69, 18. 31478576 10.1111/idj.12505 PMC9379045

[7] Ramanauskaite, A. , T. Fretwurst , F. Schwarz , Int. J. Implant. Dent. 2021, 7, 112. 34779939 10.1186/s40729-021-00388-x PMC8593130

[8] L. Larsson , A. M. Decker , L. Nibali , S. P. Pilipchuk , T. Berglundh , W. V. Giannobile , J. Dent. Res. 2016, 95, 255. 26608580 10.1177/0022034515618887 PMC4766955

[9] Z. Liu , Y. Yi , S. Wang , H. Dou , Y. Fan , L. Tian , J. Zhao , L. Ren , ACS Nano 2022, 16, 16549. 36218160 10.1021/acsnano.2c05980

[10] J. M. Heffernan , D. J. Overstreet , B. L. Vernon , R. Y. McLemore , T. Nagy , R. C. Moore , V. S. Badha , E. P. Childers , M. B. Nguyen , D. D. Gentry , F. M. Calara , W. B. Saunders , T. Feltis , A. C. McLaren , J. Biomed. Mater. Res. B Appl. Biomater. 2022, 110, 103. 34128323 10.1002/jbm.b.34894 PMC8608705

[11] X. Dai , B. C. Heng , Y. Bai , F. You , X. Sun , Y. Li , Z. Tang , M. Xu , X. Zhang , X. Deng , Bioact. Mater. 2021, 6, 2029. 33474514 10.1016/j.bioactmat.2020.12.020 PMC7787955

[12] L. Xiao , M. Feng , C. Chen , Q. Xiao , Y. Cui , Y. Zhang , Adv. Mater. 2023, 2304982. 10.1002/adma.202304982 37875431

[13] Y. Liu , N. Li , Y.‐P. Qi , L. Dai , T. E. Bryan , J. Mao , D. H. Pashley , F. R. Tay , Adv. Mater. 2011, 23, 975. 21341310 10.1002/adma.201003882 PMC3137871

[14] Y. Liu , D. Luo , X.‐X. Kou , X.‐D. Wang , F. R. Tay , Y.‐L. Sha , Y.‐H. Gan , Y.‐H. Zhou , Adv. Funct. Mater. 2013, 23, 1404.

[15] Y. Liu , D. Luo , M. Yu , Y. Wang , S. Jin , Z. Li , S. Cui , D. He , T. Zhang , T. Wang , Y. Zhou , Adv. Funct. Mater. 2019, 29, 1806445.

[16] Y. Liu , S. Liu , D. Luo , Z. Xue , X. Yang , L. Gu , Y. Zhou , T. Wang , Adv. Mater. 2019, 31, 1807082. 10.1002/adma.201807082 30620123

[17] M. Yu , D. Luo , J. Qiao , J. Guo , D. He , S. Jin , L. Tang , Y. Wang , X. Shi , J. Mao , S. Cui , Y. Fu , Z. Li , D. Liu , T. Zhang , C. Zhang , Z. Li , Y. Zhou , Y. Liu , Bioact. Mater. 2022, 10, 93. 34901532 10.1016/j.bioactmat.2021.08.024 PMC8636921

[18] I. X. Yin , J. Zhang , I. S. Zhao , M. L. Mei , Q. Li , C. H. Chu , Int. J. Nanomed. 2020, 15, 2555. 10.2147/IJN.S246764 PMC7174845 32368040

[19] S. Tang , J. Zheng , Adv. Healthcare Mater. 2018, 7, e1701503. 10.1002/adhm.201701503 29808627

[20] R. A. Bapat , T. V. Chaubal , C. P. Joshi , P. R. Bapat , H. Choudhury , M. Pandey , B. Gorain , P. Kesharwani , Mater. Sci. Eng. C Mater. Biol. Appl. 2018, 91, 881. 30033323 10.1016/j.msec.2018.05.069

[21] C. Wang , X. Jiang , H.‐J. Kim , S. Zhang , X. Zhou , Y. Chen , H. Ling , Y. Xue , Z. Chen , M. Qu , L. Ren , J. Zhu , A. Libanori , Y. Zhu , H. Kang , S. Ahadian , M. R. Dokmeci , P. Servati , X. He , Z. Gu , W. Sun , A. Khademhosseini , Biomaterials 2022, 285, 121479. 35487064 10.1016/j.biomaterials.2022.121479

[22] Z. Yang , L. Ma , X. Han , X. Xun , T. Li , K. Duan , X. Hu , Y. Wan , H. Ao , Composites, Part B 2022, 238, 109945.

[23] X. Liu , Z. Cai , M. Pei , H. Zeng , L. Yang , W. Cao , X. Zhou , F. Chen , Adv. Healthcare Mater. 2023, e2302893. 10.1002/adhm.202302893 38060694

[24] Z. Li , D. He , B. Guo , Z. Wang , H. Yu , Y. Wang , S. Jin , M. Yu , L. Zhu , L. Chen , C. Ding , X. Wu , T. Wu , S. Gong , J. Mao , Y. Zhou , D. Luo , Y. Liu , Nat. Commun. 2023, 14, 6963. 37907455 10.1038/s41467-023-42598-4 PMC10618168

[25] K. G. Heiple , V. M. Goldberg , A. E. Powell , G. D. Bos , J. M. Zika , Orthop. Clin. North Am 1987, 18, 179. 3550570

[26] W. H. De Jong , J. Eelco Bergsma , J. E. Robinson , R. R. M. Bos , Biomaterials 2005, 26, 1781. 15576152 10.1016/j.biomaterials.2004.06.026

[27] R. López‐Píriz , E. Solá‐Linares , M. Rodriguez‐Portugal , B. Malpica , I. Díaz‐Güemes , S. Enciso , L. Esteban‐Tejeda , B. Cabal , J. J. Granizo , J. S. Moya , R. Torrecillas , PLoS One 2015, 10, e0140374. 26489088 10.1371/journal.pone.0140374 PMC4619200

[28] S.‐Y. Park , K.‐H. Kim , S.‐H. Rhee , J.‐C. Lee , S.‐Y. Shin , Y.‐M. Lee , Y.‐J. Seol , Clin. Oral. Implants Res. 2017, 28, 36. 25958979 10.1111/clr.12611

[29] W. J. Seong , G. Kotsakis , J.‐K. Huh , S. C. Jeong , K. Y. Nam , J. R. Kim , Y. C. Heo , H.‐C. Kim , L. Zhang , M. D. Evans , H. Conrad , R. J. Schumacher , BMC Oral. Health 2019, 19, 150. 31307461 10.1186/s12903-019-0837-y PMC6632201

[30] A. C. McLaren , Clin. Orthop. Relat. Res. 2004, 427, 101. 10.1097/01.blo.0000143554.56897.26 15552144

[31] Y. Zhang , K. Gulati , Z. Li , P. Di , Y. Liu , Nanomaterials 2021, 11. 10.3390/nano11102489 PMC8538755 34684930

[32] S. Maher , A. Mazinani , M. R. Barati , D. Losic , Expert Opin. Drug Deliv. 2018, 15, 1021. 30259776 10.1080/17425247.2018.1517743

[33] R. Wang , M. Shi , F. Xu , Y. Qiu , P. Zhang , K. Shen , Q. Zhao , J. Yu , Y. Zhang , Nat. Commun. 2020, 11, 4465. 32901012 10.1038/s41467-020-18267-1 PMC7479592

[34] X. Li , M. Qi , X. Sun , M. D. Weir , F. R. Tay , T. W. Oates , B. Dong , Y. Zhou , L. Wang , H. H. K. Xu , Acta Biomater. 2019, 94, 627. 31212111 10.1016/j.actbio.2019.06.023

[35] X. Jiang , Y. Zhang , B. Chen , Y. Lin , Clin. Implant Dent. Relat. Res. 2017, 19, 296. 27534447 10.1111/cid.12442

[36] M.‐E. Jennes , M. Naumann , S. Peroz , F. Beuer , F. Schmidt , Antibiotics, (Basel) 2021, 10, 1350. 34827288 10.3390/antibiotics10111350 PMC8615005

[37] K. Sinjab , C. Garaicoa‐Pazmino , H. L. Wang , Implant. Dent. 2018, 27, 276. 29762186 10.1097/ID.0000000000000775

[38] L. J. Heitz‐Mayfield , N. P. Lang , Periodontol. 2000 2010, 53, 167. 20403112 10.1111/j.1600-0757.2010.00348.x

[39] C. R. Arciola , D. Campoccia , P. Speziale , L. Montanaro , J. W. Costerton , Biomaterials 2012, 33, 5967. 22695065 10.1016/j.biomaterials.2012.05.031

[40] A. S. Jain , P. S. Pawar , A. Sarkar , V. Junnuthula , S. Dyawanapelly , Int. J. Mol. Sci. 2021, 22, 11993. 34769419 10.3390/ijms222111993 PMC8584914

[41] T. Matsunaga , R. Tomoda , T. Nakajima , N. Nakamura , T. Komine , Appl. Environ. Microbiol. 1988, 54, 1330. 3046487 10.1128/aem.54.6.1330-1333.1988 PMC202658

[42] T. Xia , M. Kovochich , J. Brant , M. Hotze , J. Sempf , T. Oberley , C. Sioutas , J. I. Yeh , M. R. Wiesner , A. E. Nel , Nano Lett. 2006, 6, 1794. 16895376 10.1021/nl061025k

[43] J. N. Meyer , C. A. Lord , X. Y. Yang , E. A. Turner , A. R. Badireddy , S. M. Marinakos , A. Chilkoti , M. R. Wiesner , M. Auffan , Aquat. Toxicol. 2010, 100, 140. 20708279 10.1016/j.aquatox.2010.07.016

[44] S. Chen , A. E. Goode , S. Sweeney , I. G. Theodorou , A. J. Thorley , P. Ruenraroengsak , Y. Chang , A. Gow , S. Schwander , J. Skepper , J. J. Zhang , M. S. Shaffer , K. F. Chung , T. D. Tetley , M. P. Ryan , A. E. Porter , Nanoscale 2013, 5, 9839. 23970174 10.1039/c3nr03205a PMC4337028

[45] J. H. Ji , J. H. Jung , S. S. Kim , J.‐U. Yoon , J. D. Park , B. S. Choi , Y. H. Chung , I. H. Kwon , J. Jeong , B. S. Han , J. H. Shin , J. H. Sung , K. S. Song , I. J. Yu , Inhal. Toxicol. 2007, 19, 857. 17687717 10.1080/08958370701432108

[46] L. Zhao , H. Wang , K. Huo , L. Cui , W. Zhang , H. Ni , Y. Zhang , Z. Wu , P. K. Chu , Biomaterials 2011, 32, 5706. 21565401 10.1016/j.biomaterials.2011.04.040

[47] A. Taglietti , C. R. Arciola , A. D'Agostino , G. Dacarro , L. Montanaro , D. Campoccia , L. Cucca , M. Vercellino , A. Poggi , P. Pallavicini , L. Visai , Biomaterials 2014, 35, 1779. 24315574 10.1016/j.biomaterials.2013.11.047

[48] H. M. Yun , B. Kim , Y. H. Jeong , J. T. Hong , K. R. Park , BioFactors 2022, 49, 127. 35852295 10.1002/biof.1878 PMC10947220

[49] Z. Li , W. Wang , H. Xu , Y. Ning , W. Fang , W. Liao , J. Zou , Y. Yang , N. Shao , Am. J. Transl. Res. 2017, 9, 1680. 28469774 PMC5411917

[50] A. Liu , S. Jin , C. Fu , S. Cui , T. Zhang , L. Zhu , Y. Wang , S. G. F. Shen , N. Jiang , Y. Liu , Int. J. Oral. Sci. 2020, 12, 33. 33257654 10.1038/s41368-020-00100-6 PMC7705747

[51] B.‐M. Seo , M. Miura , S. Gronthos , P. Mark Bartold , S. Batouli , J. Brahim , M. Young , P. Gehron Robey , C. Y. Wang , S. Shi , Lancet 2004, 364, 149. 15246727 10.1016/S0140-6736(04)16627-0

