# Supplementary material for: A Biomimetic Multifunctional Scaffold for Infectious Vertical Bone Augmentation
Source: Adv Sci (Weinh). 2024 May 5;11(26):2310292. doi: 10.1002/advs.202310292 (PMC11234421; doi:10.1002/advs.202310292)
Supplement: Supplementary file 1 — Supporting Information [file ADVS-11-2310292-s001.docx]

Supporting Information

A Biomimetic Multifunctional Scaffold for Infectious Vertical Bone Augmentation

Yifan Zhang, Zixin Li, Houzuo Guo, Qibo Wang, Bowen Guo, Xi Jiang, Yishu Liu, Shengjie Cui, Zhengda Wu, Min Yu, Lisha Zhu, Liyuan Chen, Ning Du, Dan Luo, Ye Lin*, Ping Di*, and Yan Liu*


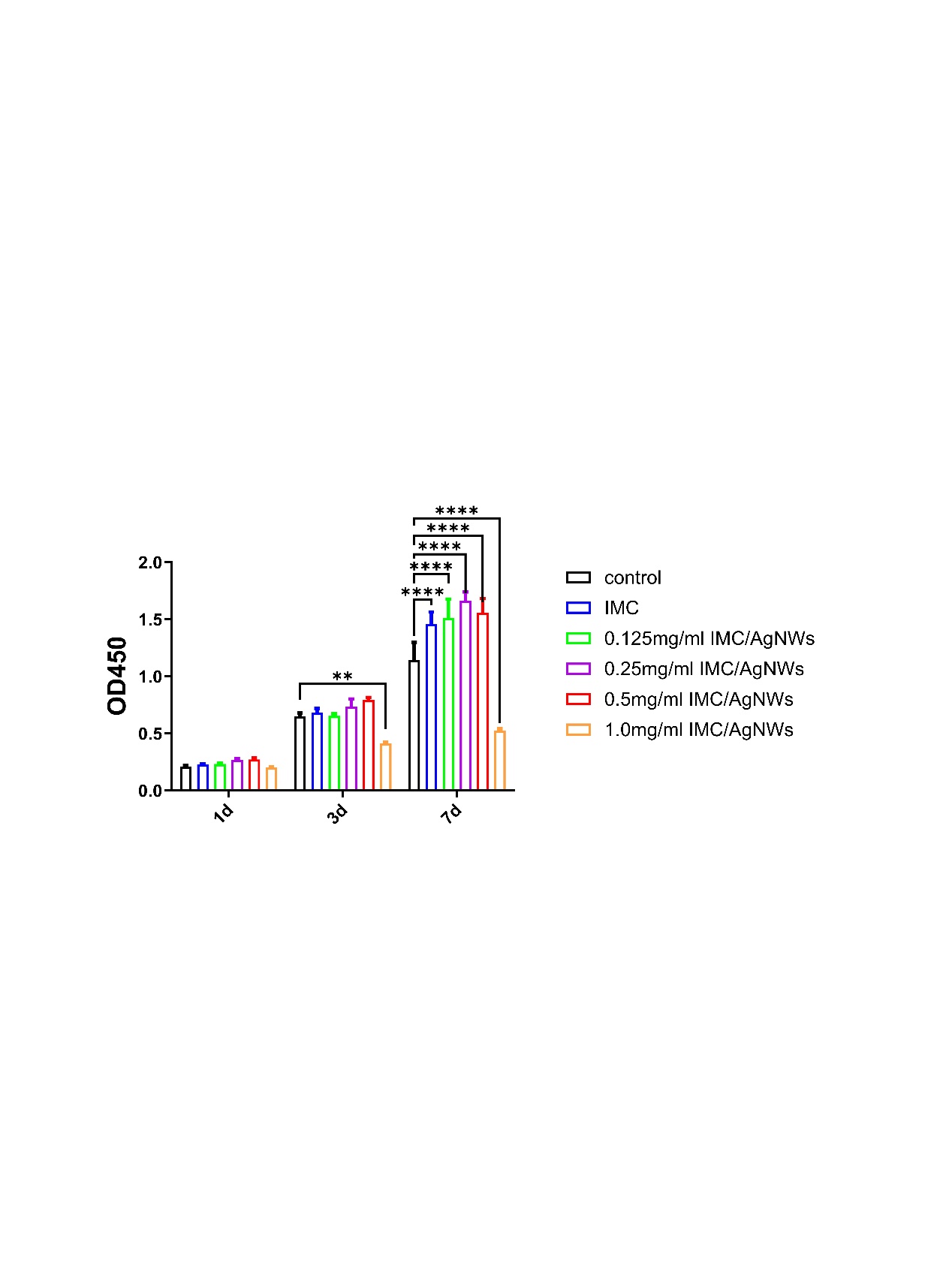
**Supplementary figures**

**Figure S1.** CCK-8 assay for screening the appropriate concentration of AgNWs, where 1.0 mg/mL IMC/AgNWs showed significantly poorer biocompatibility and potential cytotoxicity compared to the control group on days 3 and 7. *n* = 3 independent samples; **: *p*< 0.01; ****: *p*< 0.0001. Control: culture plates.


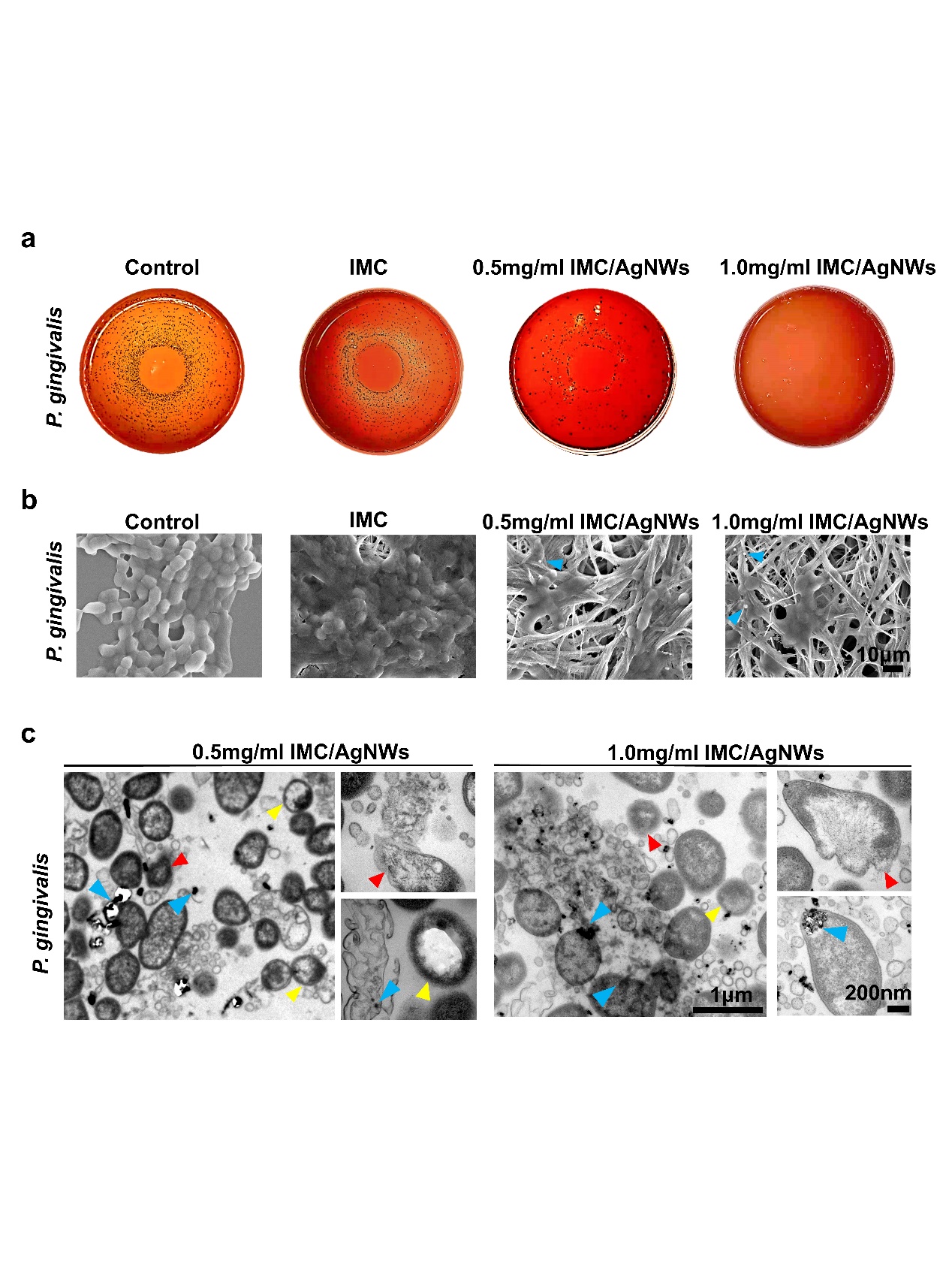


**Figure S2.** (a) Colony form units (CFU) assay of the upper suspension of *P. gingivalis* (1×10^6^ CFU mL^-1^) cultured on different substrates for 48 h. Control: culture plates. (b) SEM images of the formation of *P. gingivalis* biofilms on different substrates for 72 h. Blue arrows: Ag nanoparticles. (c) TEM images of *P. gingivalis* cultured with 0.5 mg mL^-1^ (left) and 1.0 mg mL^-1^ IMC/AgNWs (right) for 48 h. The Ag nanoparticles (indicated as blue arrows) were observed even within the *P. gingivalis.* Loss of distinctness of the cell membrane structures (red arrows) and decrease of the electron density in the cytoplasm (yellow arrows) demonstrated the dramatic ultrastructural disruption of *P*. *gingivalis*.


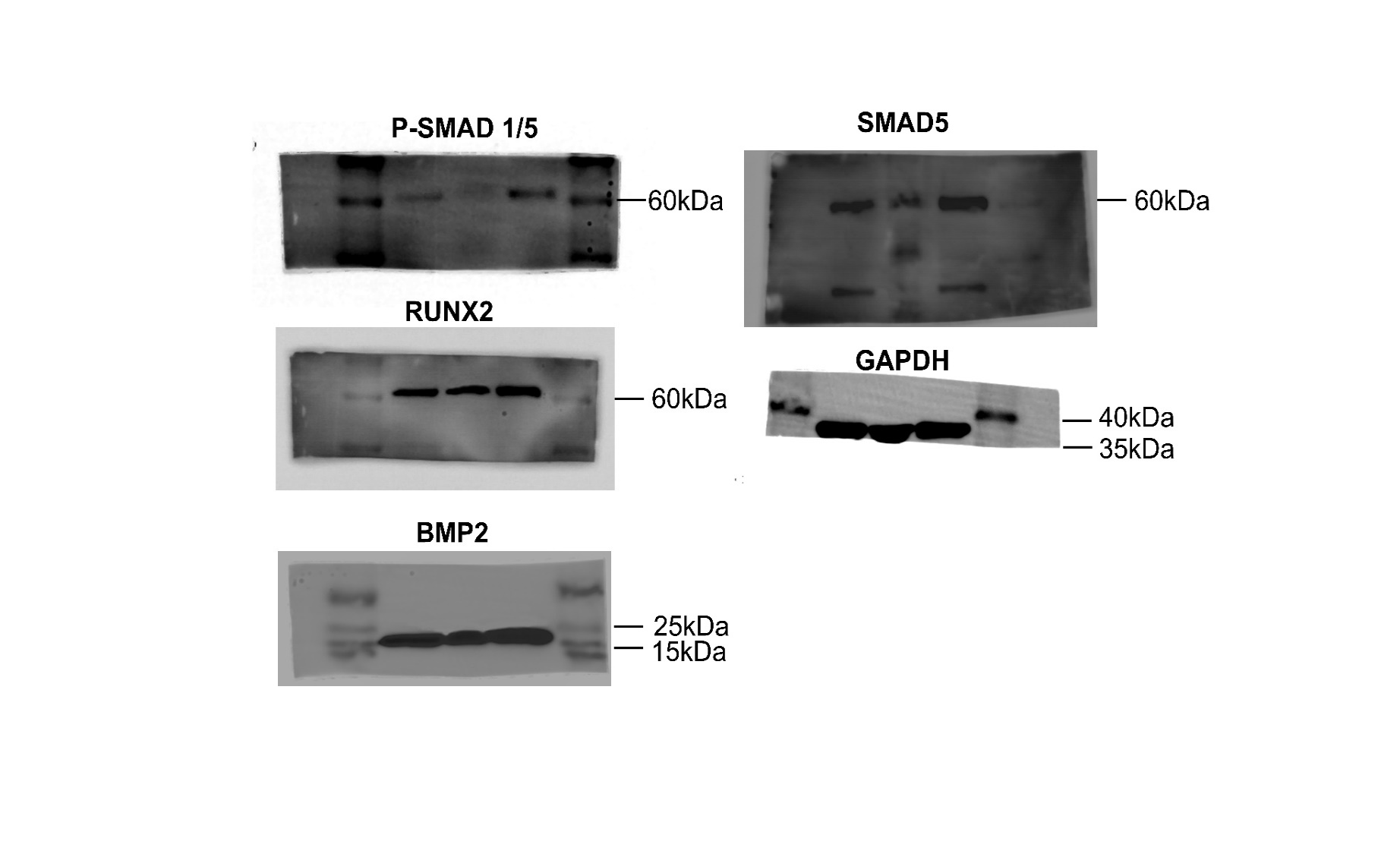
**Figure S3.** Original scans of Western blotting in Figure 3e.


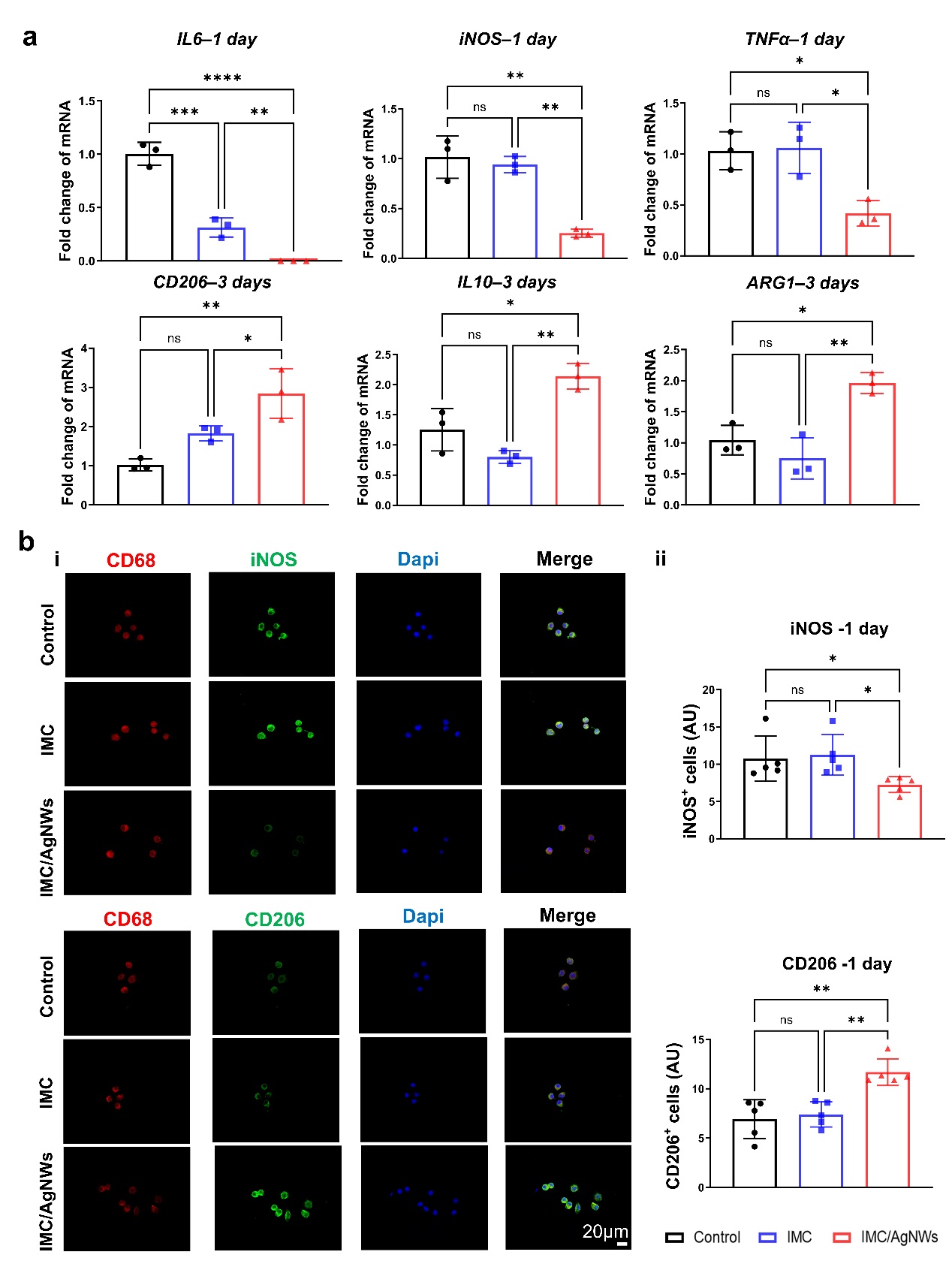
**Figure S4.** IMC/AgNWs inhibited inflammatory responses *in vitro.* (a) Relative mRNA expression of *Il6*, *Inos*, *Tnfα*, *Cd206*, *Il10*, and *Arg1* in RAW264.7 cells cultured on different substrates including culture plates (control), IMC, and 0.5mg/mL-IMC/AgNWs for 1 and 3 days. (b) (i) Immunofluorescent staining of CD68 (red), iNOS (green), and nuclei (blue) after RAW264.7 cells seeded on different substrates for 1 day. (ii) Semi-quantitation of iNOS^+^ cells and CD206^+^ cells among different groups. *: *p*< 0.05; **: *p*< 0.01; ***: *p*< 0.01; ****: *p*< 0.0001. ns: non-significant difference.

**
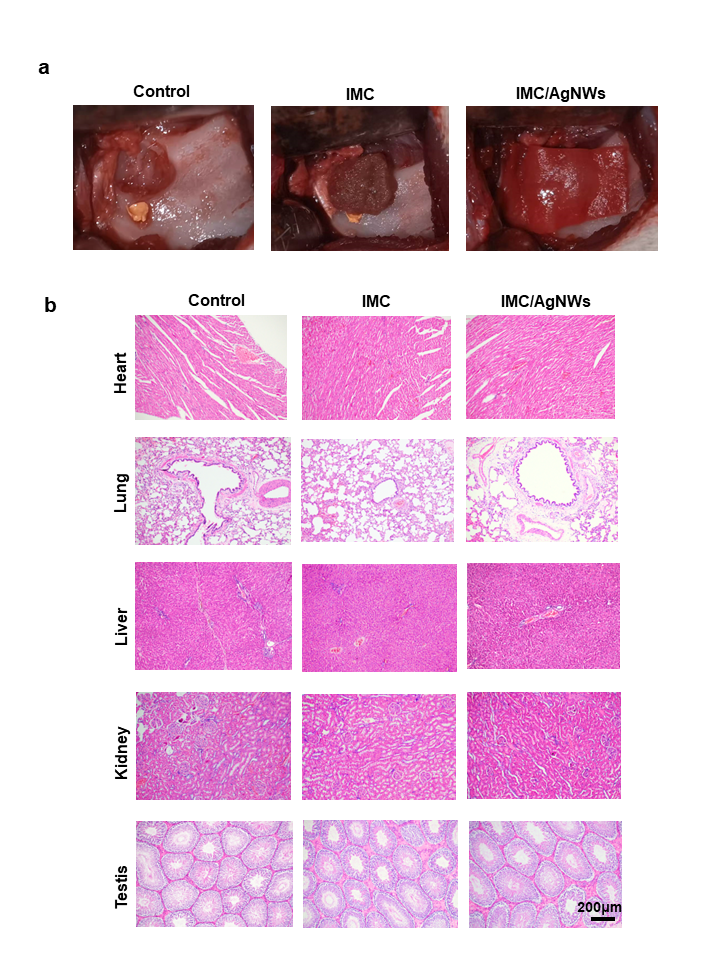
Figure S5.** (a) The surgical photos of the establishment and treatment of rat mandibular infected periodontal defects in different groups. (b) HE staining images of heart, liver, lung, kidney, and testis of rats among three groups.

**Figure S6.**
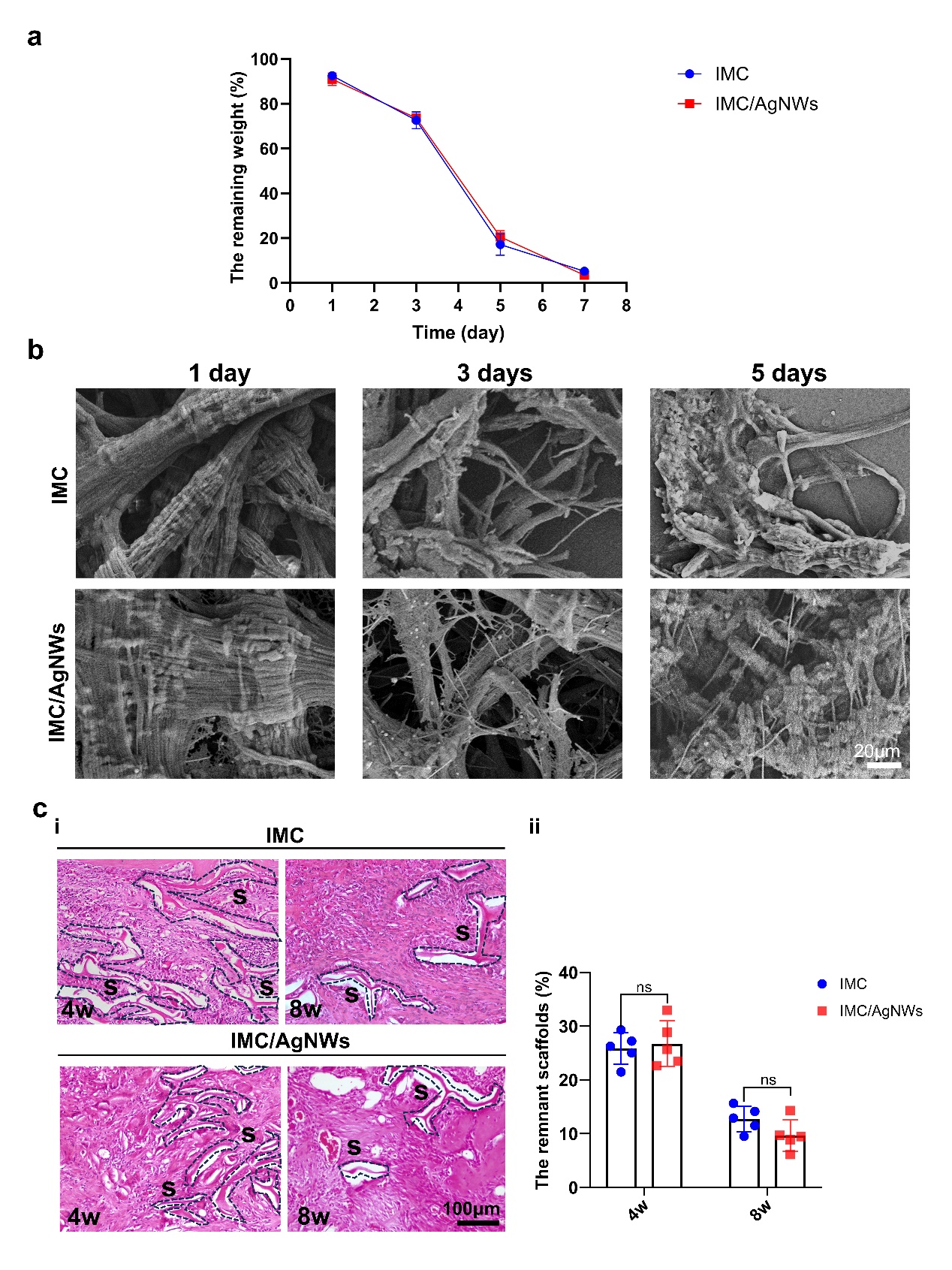
The degradation rate of the IMC/AgNWs scaffold *in vitro* and *in vivo.* (a) The remaining weight of both IMC and IMC/AgNWs scaffold in collagenase solution on days 1, 3, 5, and 7. (b) The SEM images of IMC and IMC/AgNWs after degradation in collagenase solution on days 1, 3, and 5. (c) (i) The HE staining images showed some undegraded scaffolds in the defect area at 4 weeks or 8 weeks post-implantation. (ii) Semi-quantitative analysis of the remnant scaffolds based on the HE images at 4 weeks or 8 weeks post-implantation. ns: non-significant difference; S: scaffold.

**
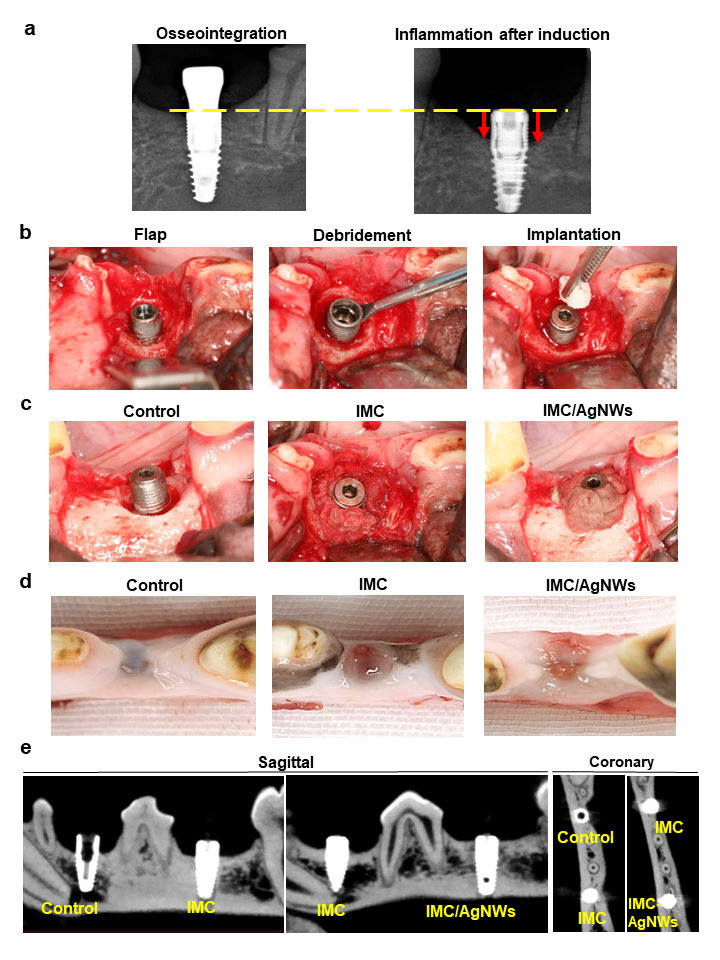

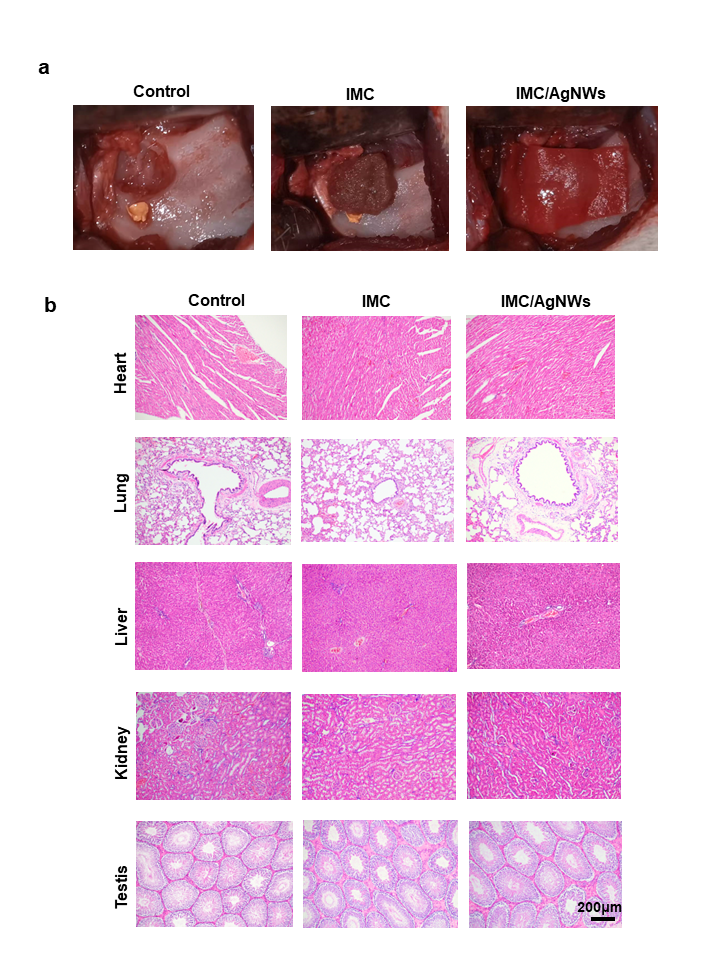
Figure S7.** (a) The successful establishment of peri-implantitis model in beagle dogs, as shown by periapical radiograph over the same implant after 3 months of inflammation induction. (b) The gross morphology of the surgical protocol of peri-implantitis treatment: flap surgery, debridement by means of Ti-brush and chemical rinse, and scaffold implantation. (c) The general view of different groups after scaffold implantation. (d) The general view of peri-implantitis sites after 3 months of treatment among different groups. (e) The sagittal and coronary CBCT images of peri-implantitis sites after 3 months of treatment, showing that IMC/AgNWs almost regenerates peri-implant alveolar defects.

**Table S1.** List of chemicals and recombinant proteins used in the study.

| **Chemicals and peptides** | | |
| --- | --- | --- |
| Penicillin-Streptomycin | Thermo Fisher Scientific | Cat#15070063 |
| Trypsin-EDTA | Hyclone | Cat#SH30042.01 |
| TRIzol Reagent | Thermo Fisher Scientific | Cat#15596026 |
| Collagen I, Rat Tail | Corning | Cat#54236 |
| Mounting Medium with DAPI | ZSGB-BIO | Cat#ZLI-9557 |
| 1-ethyl3-(3-dimehylaminopropyl) carbodiimide hydrochloride | Sigma-Aldrich | Cat#25952-53-8 |
| RIPA Buffer | Thermo Fisher Scientific | Cat#89900 |
| L-Glutamine | Thermo Fisher Scientific | Cat#25030081 |
| Fetal bovine serum（FBS） | Thermo Fisher Scientific | Cat#10099-141 |
| SYBR Green Supermix | Thermo Fisher Scientific | Cat#4385612 |
| Dexamethasone | Sigma-Aldrich | Cat#D8893 |
| TBS | Solarbio | T1080 |
| Tween | Sigma-Aldrich | P9416 |
| Sodium phosphotungstate | Aladdin | Cat#51312-42-6 |
| Poly-(α,β)-DL-aspartic acid sodium salt | Sigma-Aldrich | Cat#94525-01-6 |
| N-Hydroxysuccinimide | Sigma-Aldrich | Cat#6066-82-6 |
| Lipopolysaccharides from P. gingivalis | Sigma-Aldrich | Cat# SMB00610 |
| LB Broth | Sigma-Aldrich | Cat#L7275 |
| Bio-Gide® | Geistlich Pharma AG | Cat#20141 |
| 2′,7′-Dichlorofluorescin diacetate | Solarbio | Cat#4091-99-0 |
| Fluo 3-AM | Solarbio | Cat#121714-22-5 |
| **Critical commercial assays** | | |
| ReverTra Ace qPCR RT Kit | TOYOBO | Cat#FSQ-101 |
| Masson’s Trichrome Stain Kit | Solarbio | Cat#G1340 |
| Pierce BCA protein assay Kit | Thermo Fisher Scientific | Cat#23225 |
| Enhanced Chemiluminescence Western Blotting Detection Kit | Thermo Fisher Scientific | Cat#34577 |
| Cell Counting Kit-8 | Solarbio | Cat#1210 |
| Calcein-AM/PI Live/Dead Stain Kit | Solarbio | Cat#1630 |

**Table S2.** List of primers used in the study.

| **Gene** | **Assay** | **Forward (5’-3’)** | **Reverse (5’-3’)** |
| --- | --- | --- | --- |
| hGAPDH | qRT-PCR | GGAGCGAGATCCCTCCAAAAT | GGCTGTTGTCATACTTCTCATGG |
| hBMP-2 | qRT-PCR | TGCACCAAGATGAACACAGC | TTCCGCTGTTTGTGTTTGGC |
| hOCN | qRT-PCR | CCTTCATGTCCAAGCAGGA | GGCGGTCTTCAAGCCATAC |
| hALP | qRT-PCR | CTCCAAAAGCTCAACACCAATG | ATTTGTCCATCTCCAGCCG |
| HRUNX2 | qRT-PCR | CACTGGCGCTGCAACAAGA | CATTCCGGAGCTCAGCAGAATAA |
| mTNFα | qRT-PCR | CACGCTCTTCTGTCTACTGAACTTC | GGGCTACGGGCTTGTCACTC |
| mGAPDH | qRT-PCR | AGAAGGTGGTGAAGCAGGCATC | CGAAGGTGGAAGAGTGGGAGTTG |
| mARG1 | qRT-PCR | GAAGACAGCAGAGGAGGTGAAGAG | CAGTCCCTGGCTTATGGTTACCC |
| mIL10 | qRT-PCR | GGACAACATACTGCTAACCGACTC | TGGATCATTTCCGATAAGGCTTGG |
| mCD206 | qRT-PCR | TCTGGTGAACGGAATGATTGTGTAG | GCTTTGGTTGTAATGGATGAGTGTG |
| mIL6 | qRT-PCR | TTCTTGGGACTGATGCTGGTGAC | GTGGTATCCTCTGTGAAGTCTCCTC |
| miNOS | qRT-PCR | ATCTTGGAGCGAGTTGTGGATTGTC | TAGGTGAGGGCTTGGCTGAGTG |

**Table S3.** List of reagents or resources used in the study.

| **REAGENT or RESOURCE** | **SOURCE** | **IDENTIFIER** |
| --- | --- | --- |
| **Antibodies** | | |
| Rabbit monoclonal anti-BMP-2 | Abcam | Cat#AB214821 |
| Rabbit monoclonal anti-p-Smad1/5 | Cell signaling technology | Cat#12534 |
| Rabbit monoclonal anti-Smad5 | Cell signaling technology | Cat#12534 |
| Rabbit monoclonal anti-Runx2 | Cell signaling technology | Cat# D1L7F |
| Rabbit monoclonal anti-iNOS | Abcam | Cat#AB178945 |
| Rabbit polyclonal anti-CD206 | Abcam | Cat#AB64693 |
| Rabbit polyclonal anti-CD68 | Proteintech | Cat#28058-1-AP |
| Mouse monoclonal anti-GADPH | Proteintech | Cat#60004-1-lg |
| Mouse monoclonal anti-Actin | ZSGB-BIO | Cat#TA-09 |
| HRP-linked anti-rabbit IgG | ZSGB-BIO | Cat#ZB-2305 |
| HRP-linked anti-mouse IgG | ZSGB-BIO | Cat#ZB-2301 |
| FITC-labeled goat anti-rabbit IgG(H+L) | ZSGB-BIO | Cat#ZF-0311 |
| RBITC-labeled goat anti-mouse IgG | Solarbio | Cat# SR131 |

**Table S4.** List of animals used in the study.

| Defects | Sample size | Time points | Groups |
| --- | --- | --- | --- |
| Rat critical-sized infected periodontal defects | 30 | 4 weeks  8 weeks | Control (n = 5 at each time point)  IMC (n = 5 at each time point)  IMC/AgNWs (n = 5 at each time point) |
| Dog peri-implantitis defects | 6 (two bone defects at each side and four defects in one dog and totally 24 defects) | 12 weeks | Control (*n* = 8)  IMC (*n* = 8)  IMC/AgNWs (*n* = 8) |

**Table S5.** Software and Algorithms.

| μCT Evaluation CTAn software | <https://www.blut->scientific.com/bruker-micro-ct-software/ |
| --- | --- |
| Graph Pad Prism 8 | https://www.graphpad.com/ |
| Nanoscope analysis 1.9 | https://www.bruker.com/ |
| ImageJ/Fiji | http://fiji.sc |
